# Forecasting East Asian Indices Futures via a Novel Hybrid of Wavelet-PCA Denoising and Artificial Neural Network Models

**DOI:** 10.1371/journal.pone.0156338

**Published:** 2016-06-01

**Authors:** Jacinta Chan Phooi M’ng, Mohammadali Mehralizadeh

**Affiliations:** Faculty of Business and Accountancy, University of Malaya, Kuala Lumpur, 50603, Malaysia; Tianjin University, CHINA

## Abstract

The motivation behind this research is to innovatively combine new methods like wavelet, principal component analysis (PCA), and artificial neural network (ANN) approaches to analyze trade in today’s increasingly difficult and volatile financial futures markets. The main focus of this study is to facilitate forecasting by using an enhanced denoising process on market data, taken as a multivariate signal, in order to deduct the same noise from the open-high-low-close signal of a market. This research offers evidence on the predictive ability and the profitability of abnormal returns of a new hybrid forecasting model using Wavelet-PCA denoising and ANN (named WPCA-NN) on futures contracts of Hong Kong’s Hang Seng futures, Japan’s NIKKEI 225 futures, Singapore’s MSCI futures, South Korea’s KOSPI 200 futures, and Taiwan’s TAIEX futures from 2005 to 2014. Using a host of technical analysis indicators consisting of RSI, MACD, MACD Signal, Stochastic Fast %K, Stochastic Slow %K, Stochastic %D, and Ultimate Oscillator, empirical results show that the annual mean returns of WPCA-NN are more than the threshold buy-and-hold for the validation, test, and evaluation periods; this is inconsistent with the traditional random walk hypothesis, which insists that mechanical rules cannot outperform the threshold buy-and-hold. The findings, however, are consistent with literature that advocates technical analysis.

## Introduction

Algorithmic trading has evolved exponentially in recent years due to more rapid reactions to temporary mispricing and easier price management with computational trading systems, which can learn from thousands of information sources without the hindrance of human emotions [1, 2]. Technical analysis, the methodology and science of deciphering past historical data to forecast future prices, has also grown to include machine learning methods, like the Artificial Neural Network (ANN) approach [3].

Futures markets possess a fluctuating and volatile nature, which makes them appealing to a variety of people for different reasons, including investors who are attracted to them because of high returns and researchers who are eager to model market trends as an organized complexity. Advocates of the Random Walk (RW) and Efficient Market Hypothesis (EMH) approaches argue that financial markets are not predictable, based on current and historical data [4, 5]. However, there are many studies that oppose this view and argue in favor of the predictability of financial time series [6, 7].

The purpose of this research is therefore twofold: it not only tests and develops innovative uses of Wavelet Principal Component Analysis (WPCA) and ANN on time series, but also offers to trading practitioners a timely trading method to make better trading decisions in financial markets where studies show that abnormal returns using basic technical indicators of yesteryear are fast declining [8]. Coronel-Brizio et al. [9] find empirical evidence that financial markets are evolving and increasing their efficiency over time. With increasing efficiency, abnormal profits are harder to come by [10]. Since traditional statistical methods have reached their limitations [11, 12], machine learning systems are currently used in forecasting financial time series. ANN, as a capable prediction tool [13], outperforms traditional statistical methods and various other intelligent models [14–16]. With the capability of ANNs to establish complex relationships between training variables and targets, they improve the chances to predict highly complicated and volatile trends in the markets [17–19]. Bahrammirzaee [12] argues that researchers use ANN due to its qualities such as efficiency, performance, reproducibility, consistency, completeness, breadth, and consistency of decision making, and most importantly, timeliness. However, Guo et al. [20] and Chang et al. [21] believe ANNs are limited due to the complexities of the chaotic behaviors associated with financial markets, the multivariate signals emitted by the time series, the risk of model over-fitting, and the noise in time series.

Guo et al. [22] and Taha [23] propose that feature selection techniques be used to combine different machine learning methods into a new effective learning system that assembles the best-performing and strongest features of each approach, while leaving out the defects and weak points; they argue that such a synthesis can provide a better representation of machine-trading systems with the ability to process and learn within both non-symbolic and symbolic paradigm. Lertpalangsunti and Chan [24] offer three general reasons for introducing hybrid models, these being technique improvement, diversity of application duties, and recognition of multi-functionality. Hence, ANNs can be used with other machine-learning models in parallel, transformational, or sequential methods, to overcome their limitations and deficiencies. In this context, studies such as [25–30] are suggested for more details.

Due to the vulnerability of ANNs to futures price noises, wavelet analysis is applied in combination with ANN in this study. Wavelet decomposition (Wavelet Transform) is a technical tool for analyzing signals, employed for its superiority in evaluating signals in two main domains: frequency and time [31]. Denoising algorithms based on Wavelet Transform have become a widespread technique for single-dimensional signal filtering and data mining [28, 32, 33]. Several studies have proved that Wavelet Transform denoising procedures improve the performance of time series forecasting [34, 35]. Hsieh et al. [28] propose an ensemble system in which wavelet denoising and a recurrent neural network are combined to forecast DJIA, FTSE, NIKKEI, and TAIEX with promising profitable trading results. Lotrič and Dobnikar [36] and Lotrič [37] combine the wavelet denoising method with ANN to optimize the denoising factors dynamically; they report performance improvement in forecasting accuracy. Moazzami et al. [38] use Wavelet Transform along with ANN to predict the day-ahead peak load of Iran’s national grid and show successful outcomes. Jin and Kim [39] propose some hybrid models of wavelet approximation, autoregressive integrated moving average (ARIMA), generalized autoregressive conditional heteroscedastic (GARCH), and ANN, to predict natural gas prices. The results show not only that the performance of the wavelet combination is superior in all models, but also wavelet-ANN outperforms other cases. There have been studies on wavelet and neural networks, where wavelets are applied in hidden layers, and as neuron transfer functions, called “wavelet networks.” [14, 17] Instead of employing wavelet coefficients in training the neural network, this study uses Wavelet Transform for denoising signals and then feeds them into the neural network.

As wavelet denoising is a univariate algorithm, Aminghafari et al. [32] offer a multivariate denoising using wavelets and principal component analysis (PCA). PCA is one of the best-known data analysis techniques, especially designed to simplify multiscale signals by tracing new factors and obtaining the main features of data [40]. Wavelet PCA (WPCA) analyzes multivariate signals with multiple univariate wavelets and then performs a PCA in order to select a convenient number of useful principal components. Aminghafari et al. [32] believe that denoising multivariate signals by Wavelet PCA (WPCA) outperforms univariate wavelet denoising on each factor separately, whereas Wavelet PCA (WPCA) extracts the same noise at different frequencies from factors of a multivariate signal.

From the literature, it is apparent that wavelet decompositions (Wavelet Transforms) can control noise in a signal, while ANN can learn various movements in nonlinear time series. Gao et al. [41–44] established a novel analytical framework of multivariate complex networks and time-frequency representation to investigate the nonlinear dynamical behavior underlying time series. Generally, hybrid models are developed in three stages, these being preprocessing, modeling, and evaluation. In this study, we propose a novel forecasting approach, which integrates WPCA and the nonlinear autoregressive ANN with exogenous input (NARX-NN) in the preprocessing stage to develop an ensemble forecasting model, a Wavelet PCA Neural Network (WPCA-NN) embedded with a trading strategy. The NARX Neural Network (NARX-NN) is a type of recurrent dynamic neural network, with feedback links attached to some layers of the network [45, 46]. The contribution of this study to the existing literature and practice will be that it is the first attempt to apply WPCA to denoise the Open-High-Low-Close (OHLC) index as a multivariate signal in order to feed to a NN to forecast future prices. We believe that through this solution, where we analyze the OHLC as a multivariate signal, there is an opportunity to extract the common noise components of these four signals more accurately. Therefore, the proposed model, Wavelet PCA with Neural Network (WPCA-NN), can capture more appropriate inputs compared with the neural network (NN) and wavelet neural network (WNN) approaches. In this experiment, three different models, NN [45, 46], WNN [18], and WPCA with Neural Network (WPCA-NN), are set up to be evaluated against not only the threshold buy-and-hold strategy [5], but also against each other for predictive accuracy and trading performance results.

To evaluate the performance of these models, this research tests these trading systems in five East Asian markets, namely Hong Kong’s Hang Seng Futures, Japan’s NIKKEI 225 futures (NIKKEI), Singapore’s MSCI futures (SiMSCI), South Korea’s KOSPI 200 futures (KOSPI), and Taiwan’s TAIEX futures (TAIEX). Apart from Japan’s NIKKEI 225 futures market, these are the futures markets of the Asian Tigers’ economies, for which studies show rapid growth, not only in monetary terms, but also in importance to the current world economy in the global trend towards diversification [47–49]. The sample data of this study are collected from Bloomberg and consist of 10 years’ worth of OHLC and popular technical indicators, RSI, MACD, MACD Signal, Stochastic Fast %K, Stochastic Slow %K, Stochastic %D, and Ultimate Oscillator.

While most studies concentrate only on the error terms of the forecasting accuracy [11, 12], this study demonstrates the possibility of accurate prediction of market direction by examining the profitability of the WPCA-NN model against the threshold buy-and-hold, NN, and WNN. Hence, a trading strategy of buying when the predicted value is higher than the current market close, and selling if otherwise, is applied on these five East Asian futures markets.

This paper should interest market traders who, in today’s increasingly difficult and volatile markets, find that basing their trading decisions solely on traditional technical analysis signals is not as profitable as it used to be. The empirical results of this research are in support of previous academic literature [11, 12] that provides evidence of successfully forecasting future price movements using machine learning methods like ANNs, genetic programming, and wavelet analysis.

The remainder of this study is organized as follows. Section 2 gives a brief introduction to multivariate denoising using wavelet and PCA as well as ANNs. In section 3, we state the overall design of this experiment and the proposed hybrid models of forecasting. Section 4 presents and discusses the prediction and returns results for the three proposed models on the five futures markets. Section 5 reports a summary of the results and concludes the study.

## Denoising and Forecasting Methods

According to the literature, ANNs and Wavelet Transforms have been successful in many cases of financial markets forecasting both in single and hybrid forms [12]. This research first denoises the historical data of open, high, low, close, and technical indicators [11] using a multivariate Wavelet-PCA denoising technique and then applies the resultant series in a NARX neural network.

### Wavelet Principal Component Analysis Denoising

The fundamental goal of denoising is to remove the noise while maintaining the main data features. In recent years, the wavelet denoising technique has outperformed many traditional methods like exponential smoothing filter, moving average filter, simple nonlinear noise reduction, and linear Fourier smoothing, because it does not consider homogeneous error structures and generates more accurate information in the denoised time series with respect to the original signal than other signal analyses [50]. Hence, wavelet denoising algorithms have become a highly popular technique for single-dimensional signal filtering and mining.

The principal component analysis (PCA) technique, a competent feature extraction tool, is extensively applied in statistics, signal processing, and neural computing [51]. The basic concept in PCA is to discover the components that describe the maximum value of variance obtainable from a data vector with *L* dimensions by *P* linearly transformed components, using the mathematical technique of eigenanalysis. The essential goal in PCA is to reduce the dimensions of the data. It can be demonstrated that the treatment given by PCA is an optimal method of decreasing linear dimensionality in the mean-square evaluation [51]. Such a diminution in dimension has significant benefits. First, the computation required in further processing is decreased. Second, noise can be deducted and the significant underlying function identified. PCA can also simplify multiscale signals by tracing new factors obtained from the main features of data [40]. Aminghafari et al. [32] describe univariate, multiple one-dimensional, and multivariate wavelet denoising as follows, which procedures are also provided in the Matlab library as a coded function named “wmulden.” [52]

The simplest classical univariate wavelet denoising model is of the following form:

X(t)=f(t)+ε(t),t=1,…,n (1)

where,

(*X*(*t*))_1≤*t*≤*n*_: Observed signal;

(*ε*(*t*))_1≤*t*≤*n*_: Centered Gaussian white noise of unknown variance σ^**2**^;

*f* ∈ *L*^2^: Unknown function to be recovered from the observation according to a given orthogonal wavelet transform ((*ϕ*_*j*,*k*_)_*k*∈*Z*_, (*ψ*_*j*,*k*_)_1≤*j*≤*J*,*k*∈*Z*_), where *ϕ* is the associated scaling function, *ψ* a mother wavelet, and *J* is an appropriately selected decomposition level; and where $g_{j,k}\left(x\right)=2^{-\frac{j}{2}}g(2^{-j}x-k)$ wavelet denoising is applied in three stages:

Stage 1. Decompose the observed signal by wavelet up to level *J*;
Stage 2. Threshold the wavelet detail coefficients suitably;
Stage 3. Rebuild a denoised form of the initial signal, from the thresholded detail coefficients and the estimated coefficients, and then apply the inverse form of Wavelet Transform.

#### Multiple Univariate Wavelet Denoising

The first denoising procedure of this research is a direct generalization of the one-dimensional technique. The technique rests on a modification of the procedure followed by a standard one-dimensional soft-thresholding approach. Let us consider this *p*-dimensional model:

X(t)=f(t)+ε(t),t=1,…,n (2)

where *X*(*t*), *f*(*t*), and *ε*(*t*) are as previously defined and of size 1 × *p*. In this equation, *ε*(*t*) is a centered Gaussian white noise function with unknown covariance matrix *E*(*ε*(*t*)^*T*^*ε*(*t*)) = Σ_*ε*_. Each element of *X*(*t*) is of the previously mentioned form (1) and:

$$X^{i}\left(t\right)=f^{i}\left(t\right)+\varepsilon^{i}\left(t\right),t=1,\ldots \;,n$$ (3)

where 1 ≤ *i* ≤ *p*, and *f*^*i*^ is of some functional space like *L*^2^. Then Σ_*ε*_ as a covariance matrix, which is assumed to be positive definite, obtains the stochastic relationships among the elements of *X*(*t*).

The following stages describe multiple one-dimensional denoising, with *p* original signals (the column of *X*(*t*)) with *n* dimensions presented as an *n* × *p* matrix *X*.

Stage 1. Execute the wavelet decomposition at level *J* per signal as each column of *X*.
Stage 2. State $\hat{\Sigma_{\varepsilon }}$ as an estimator of Σ_*ε*_ and then calculate a matrix *V* such that $\hat{\Sigma_{\varepsilon }}=V\Lambda V^{T}$, where Λ = *diag*(*λ*_*i*_, 1 ≤ *i* ≤ *p*). Change the basis *D*_*j*_*V*, 1 ≤ *j* ≤ *J*, and then apply the *p* univariate threshold strategies using the threshold $t_{i}=\sqrt{2\lambda_{i}\text{log}\left(n\right)}$) for the *i*-th column of *D*_*j*_*V* to each detail. In addition to this, Donoho [33] states a list of strategies that can be applied as threshold at this stage.
Stage 3. Rebuild the denoised matrix $\overset{ˇ}{X}$ by inverting the wavelet transform, from the estimation matrices and the simplified details.

Hence, this direct generalization and parallelization of the univariate wavelet denoising over time and space (change of basis) acts first to change the basis in order to reduce the correlations among the *p* signals, and second, to employ *p* univariate wavelet denoising.

#### Multivariate Denoising Using Wavelet and Principal Component Analysis (WPCA)

Aminghafari et al. [32] employ the multiscale PCA denoising proposed by Bakshi [40] in order to develop a generalized multivariate wavelet denoising approach. The introduction of a PCA stage can take advantage of the deterministic links among the signals, offering an extra layer of denoising by omitting insignificant principal components. The multiple univariate denoising previously discussed can be generalized by focusing on the deterministic links among the ***p*** signals.

A natural way to take the deterministic links among the *p* signals into account is first to use a threshold strategy, including a change of basis, employing *V* for the details; and second, to apply a PCA by choosing the appropriate number of elements for the approximation. More accurately, the following is the general procedure for multivariate denoising:

Stage 1. Apply the level *J* wavelet decomposition of each column of *X*;
Stage 2. State $\hat{\Sigma_{\varepsilon }}$, the estimator of Σ_*ε*_ as the noise covariance matrix, equivalent to the Minimum Covariance Determinant estimator (MCD) proposed by Rousseeuw [53], applied to *D*_1_; and then calculate matrix *V* in such a way that $\hat{\Sigma_{\varepsilon }}=V\Lambda V^{T}$, where Λ = *diag* (*λ*_*i*_, 1 ≤ *i* ≤ *p*). Change the basis *D*_*j*_*V*, 1 ≤ *j* ≤ *J*, and then perform the *p* univariate thresholding strategies employing a threshold like $t_{i}=\sqrt{2\lambda_{i}\text{log}\left(n\right)}$ to each element of the *i*-th column of *D*_*j*_*V*. Moreover, Donoho [33] presents a list of strategies that can be applied as threshold at this stage:
Stage 3. Apply the PCA of the matrix *A*_*J*_ and then choose the convenient number *p*_*J*+1_ of principal components;
Stage 4. Rebuild the denoised matrix $\overset{ˇ}{X}$ by inverting the wavelet transform, from the estimation matrices and the simplified details.

The Kaiser criterion can be employed to select those components with matching eigenvalues larger than the mean of all the eigenvalues [54]. Moreover, there are some variables and settings required for the Wavelet and PCA techniques such as wavelet type, level of denoising, thresholding strategies, and selecting the number of principal components, which will be discussed later.

### Nonlinear Autoregressive Neural Network with Exogenous Inputs (NARX-NN)

Neural Networks are employed due to their advantages such as their numeric nature, the absence of data distribution assumptions, the ability to insert new data or update inputs into a trained network, and their free model estimator nature [12, 25–28, 55].

**Fig 1:**
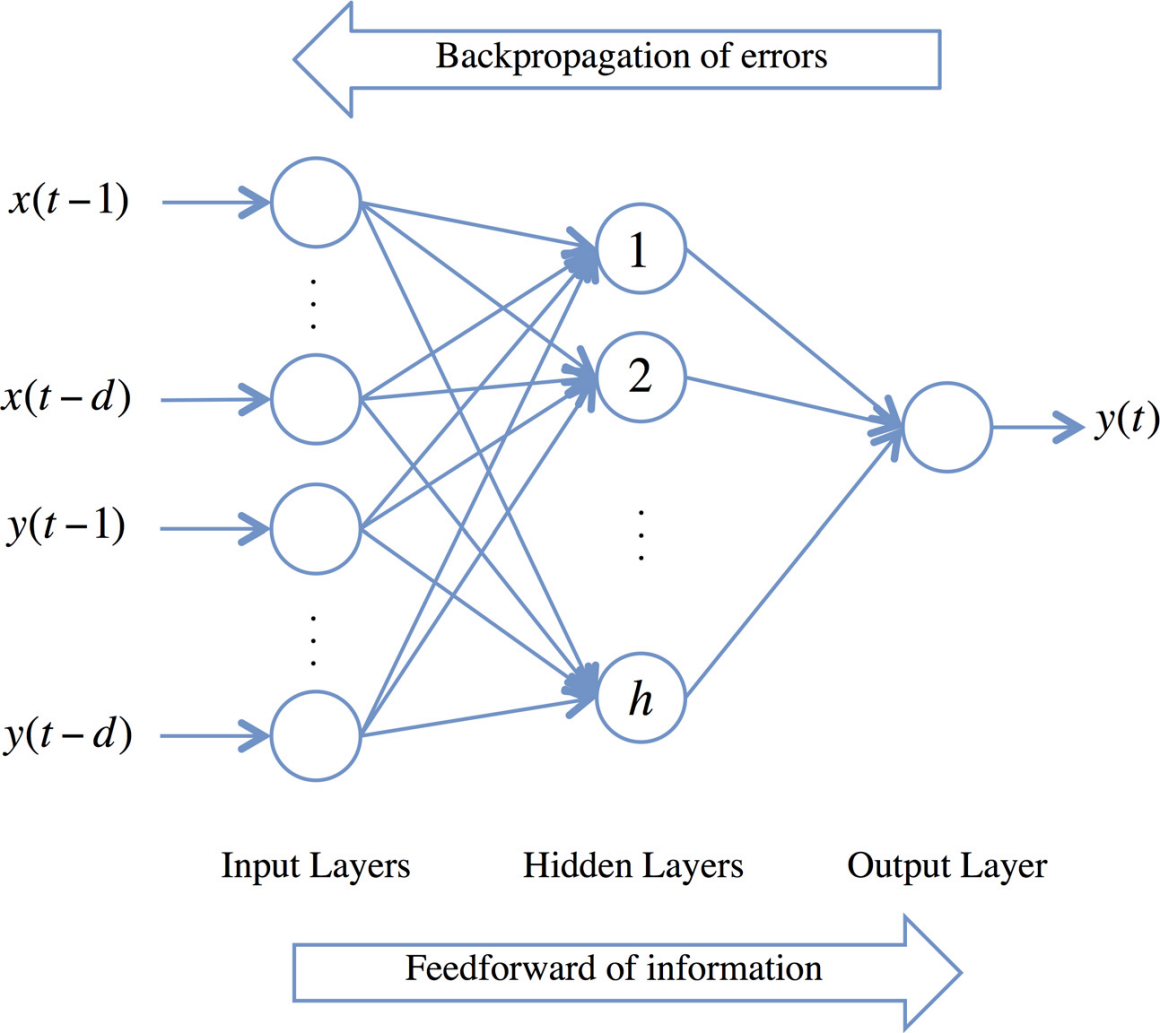
Feedforward Backpropagation Neural Network architecture.

An artificial neural network is a set of interconnected simple processing factors. Each connection of the neural network gets a weight attached to it. The Feedforward with Backpropagation Neural Network algorithm appears as one of the most broadly used machine learning techniques for multi-layer networks [56]. The standard Feedforward Backprogation Neural Network generally contains an input layer, several hidden layers, and an output layer, as displayed in Fig 1. The elements in the network are linked in a feedforward style. The weights of the links have been set as initial values. The error term between the actual value and the predicted output value is backpropagated across the network for the weights to be revised in order to minimize the error between the predicted and the actual value.

Nonlinear Autoregressive Neural Network with Exogenous Inputs (NARX-NN) is a kind of recurrent dynamic neural network with feedback links connecting some layers of the network [46]. The NARX model is built on the linear Auto Regressive Exogenous (ARX) method, which is generally applied in time series modeling.

The fundamental equation for the NARX model is:

y(t)=f(y(t−1),y(t−2),…,y(t−d),x(t−1),x(t−2),…,x(t−d)) (4)

where the obtained value of the dependent output signal *y*(*t*) is regressed on *d* former values of the target signal *y*(*t*) and *d* previous values of exogenous (independent) input signals *x*(*t*). One can implement the NARX model by applying a feedforward and backpropagation neural network (FBNN) to estimate the function *f*. Moreover, weights and biases in an FBNN will be adjusted continuously to minimize the error term between output (y*(*t* + 1)) and target value (*y* (*t* + 1)) to achieve the lowest mean of the error terms.

There are many applications for the NARX network, one of the more important being the modeling of nonlinear dynamic systems. A neural network that is considered as a learning machine system applies input series and output series of *d* previous data points to predict the next output and train itself by making the comparison between the predicted output and the actual data of the time in question. This procedure will be continuously performed, step by step and along the time series, in order to achieve the lowest mean error between the network output and the target.

In this study, a clear and efficiently coded tool in Matlab named “narxnet” [57] is used to establish a one-step-ahead prediction model. The architecture of a NARX network includes the number of hidden layers, the number of delays (the number of past data of that network that account for training), and portions of training, validation, and testing. NARX networks divide the data into three subsets: Training set, Validation set, and Testing set, which sets will be spread randomly along the time series, with a configured percentage for each of them; in this study, the proportions are training 80%, validation 10%, and testing 10%. Although the best architecture to apply depends on the type of the problem to be solved by the network, there is no rule of thumb to select the number of hidden layers and delays [19, 58]. In this study, Levenberg-Marquardt optimization is used as the training algorithm, which is a built-in algorithm in Matlab [59].

## Research Framework

This paper attempts to predict futures prices, on the basis of daily historical prices, along with their technical indicators: RSI, MACD, MACD Signal, Stochastic Fast %K, Stochastic Slow %K, Stochastic %D, and Ultimate Oscillator. The main aspect of this paper is to study the performance of the novel Wavelet PCA Neural Network (WPCA-NN) model on the futures of the Hong Kong, Japanese (NIKKEI-225), Singaporean (SiMSCI), South Korean (KOSPI-200), and Taiwanese (TAIEX) indices. The results of WPCA-NN are evaluated with those of NN and WNN and the threshold passive buy-and-hold across these East Asian futures markets to check the signal accuracy and trading profitability performance. An interesting by-product of this study is the extraction of the best setting combinations of WPCA-NN in each of these futures markets for further trading purposes.

### Data

The sample data of this study are collected from Bloomberg L.P. and consist of 13 years of historical data and seven technical indicators (3,224 daily data items for each market). The daily Open, High, Low, and Close (OHLC) as well as the Volume of Hang Seng Futures, KOSPI 200 Futures, Nikkei 225 Futures, SiMSCI Futures, and TAIEX Futures are collected from January 2, 2002, to December 31, 2014. The daily OHLC data alongside their technical indicators, RSI, MACD, MACD Signal, Stochastic Fast %K, Stochastic Slow %K, Stochastic %D, and Ultimate Oscillator are used as inputs to the models.

**Fig 2:**
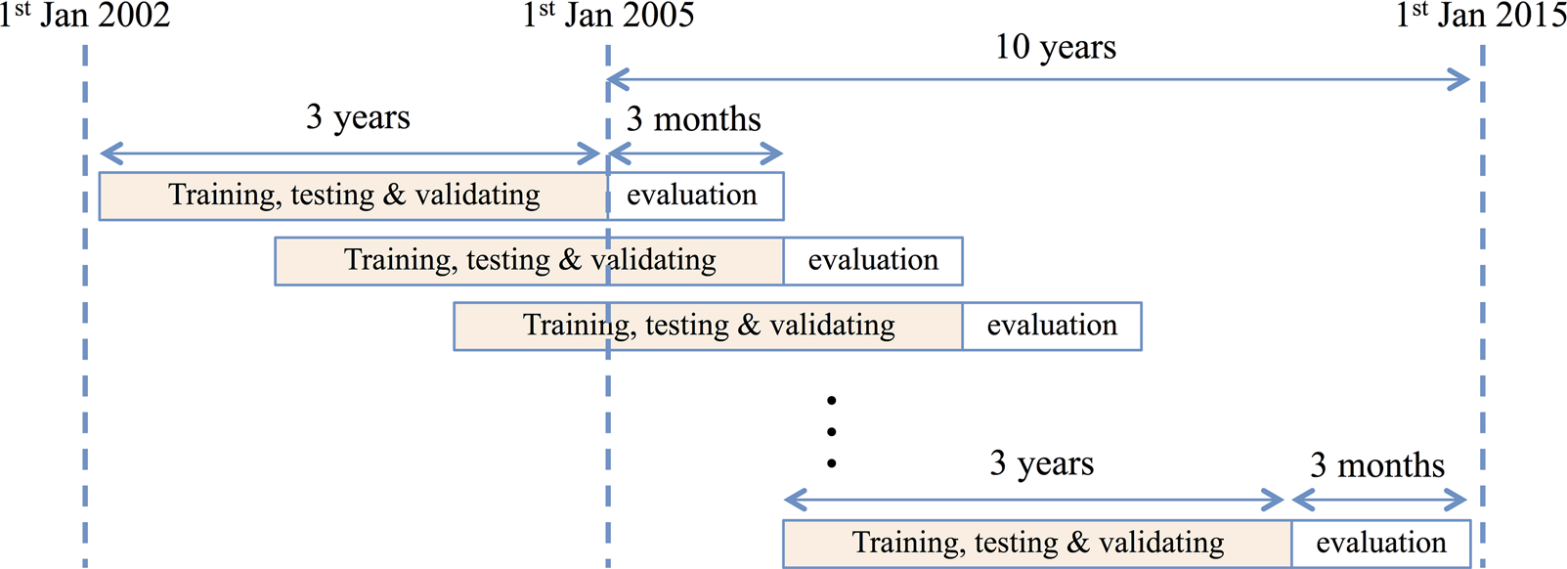
Continuously datasets arrangement for training and evaluation, 2005–2014.

The method uses the past 3 years’ (first part) worth of data of daily prices and technical indicators to forecast the following 3 months’ (second part) daily closing prices, as illustrated in Fig 2. This period of 3 years includes 80% training, 10% validating, and 10% testing. More years of training-validating-testing and a higher proportion of training (80%) may cause over-fitting problems—the memorizing of patterns by networks—thus reducing the generalizability of models [28]. The following period of 3 months (second part) is considered the evaluation period, where each of the three techniques’ (NN, WNN and WPCA-NN) performance in terms of Mean Absolute Percentage Error (MAPE) and profitability of trading strategies (Return) are measured each quarter and compared against the buy-and-hold and against each other. In line with popular portfolio management practice, quarterly evaluation is performed in this study. This process continues for 10 years on each quarter to find the performance of MAPE and the returns of the proposed techniques. Therefore, 40 quarters of five future markets from 2005 to 2014 are studied to measure the performance and compare the robustness, as well as to ensure the generalizability and practicality of our method.

We adopt the method used by [18] to address missing data; when there are data missing on some days in the original time series due to public holidays, an average of the past five days is employed to fill in the missing data point:

$$x_{t}=\frac{x_{t-1}+x_{t-2}+x_{t-3}+x_{t-4}+x_{t-5}}{5}$$ (5)

In order to check the predictability of the financial time series, it is necessary to perform the unit-root test, Johansen cointegration test, serial correlation test, and error correction model, which are offered by Fama [4] and Taylor [7]. The trend and intercept value should be strongly negative to reject the hypothesis of the unit root. The descriptive analysis shows that all five financial time series are non-stationary in the level form, but stationary in the first differenced form (S1 Table). The Johansen cointegration test is conducted in the first difference form; both trace statistics and max-eigen statistics show that at the 5% confidence level, there is cointegration for all futures markets. In other words, the historical prices move in trend and have long-term relationships with the current prices (S2 Table). Error correction terms for all futures markets are negative and significant at the 5% confidence level, which implies a long run relationship between previous data and current data in those markets (S3 Table). In finance, serial correlation is used by technical analysts to determine how well the past price of a security predicts the future price. Descriptive analysis shows serial correlation exists between previous prices and current price in all selected futures markets, at a confidence level of 10% (S4 Table).

### Model Inputs

Atsalakis and Valvanis [11], in a survey of forecasting approaches, indicate that technical analysts typically use indicators to forecast future prices. According to their study and Bahrammirzaee [12], the key types of technical indicators used to forecast financial time series are Relative Strength Index (RSI), Moving Average Convergence Divergence (MACD), MACD Signal (MACDSig), MACD Histogram (MACDHis), Stochastics (fast %K, slow %K, and %D), and Ultimate Oscillator (UO). These indicators are derived from the Open, High, Low, Close, and trading volume of the futures prices as follows:

$$RSI=100-\frac{100}{1+\frac{\sum Gainsoverthepast\;14\;periods}{\sum Lossesoverthepast\;14\;periods}}$$ (6)

MACD=(Averageofthepast12periods−Averageofthepast26periods) (7)

MACD Signal=Average of the MACD of the past8periods (8)

MACDHistogram=MACD−Signal (9)

$$Stochastic\;Fast\;\%K\;=\;100*\frac{C_{t}-Thelowestlowofthepast\;14\;periods}{ThehighestHofthepast\;14\;periods-ThelowestLofthepast\;14\;periods}$$ (10)

StochasticSlow%K=AverageoftheFast%Kofthepast3periods (11)

Stochastic%D=AverageoftheSlow%Kofthepast3periods (12)

$$UltimateOscillator\;=\;100*\frac{4*\left(\frac{BP(7)}{TR(7)}\right)+2*\left(\frac{BP(14)}{TR(14)}\right)+\frac{BP(28)}{TR(28)}}{7}$$ (13)

Where

H=Highestindexintheperiod(oneweek)

L=Lowestindexintheperiod,

C=Closingindexintheperiod

$$BP(i)\;=\;BuyingPressure(i)\;=\;\sum_{n=t-i}^{t}C_{n}-Min(C_{n-1},L_{n})$$ (14)

$$TR(i)\;=\;TrueRange(i)\;=\;\sum_{n\;=\;t-i}^{t}Max(C_{n-1},H_{n})-Min(C_{n-1},L_{n})$$ (15)

We use the same nonlinear analysis of NARX neural network and backward elimination technique [18] to select the best set of technical indicators. We key in the trading volume and these technical indicators as inputs to train the network in order to measure its performance by mean absolute percentage error (MAPE) over 10 years of each selected futures market. Therefore, we obtain a MAPE for each futures market for the trained network with selected indicators. Each time, we omit one of the indicators and retrain the network to check whether the performance (MAPE) will increase or fall, as a backward elimination technique. We compare all possible network performances in all selected markets to see which ones accurately determine their trend. The results show that presence of OHLC, RSI, MACD, MACD Signal, Stochastic Fast %K, Stochastic Slow %K, Stochastic %D, and UO are significant as inputs to the models, whereas MACD Histogram and Volume are not relevant at all for the purpose of achieving the best performance for the proposed models.

The valid inputs, namely OHLC, RSI, MACD, MACD Signal, Stochastic Fast %K, Stochastic Slow %K, Stochastic %D, and UO are then trained in neural networks to estimate the future market values. Three different models, NN, WNN, and WPCA-NN, are examined to find the best model architecture to forecast Hang Seng Futures, KOSPI Futures, Nikkei Futures, SiMSCI Futures, and TAIEX Futures. Each of these models is evaluated quarterly over 10 years (January 1, 2005, to December 31, 2014), a total of 40 datasets per market. Each dataset consists of the past 3 years for training and the current quarter for predictive accuracy and profitability performance.

One of the main focuses of this study is to denoise the multivariate OHLC signal with Wavelet-PCA. The proposed model handles multivariate signals such as OHLC. Since the other technical indicators are univariate signals, denoising them requires different methodologies, such as the discrete wavelet transform (DWT) approach [18], the use of which may contribute to a future study.

### Architecture of the Models

**Fig 3:**
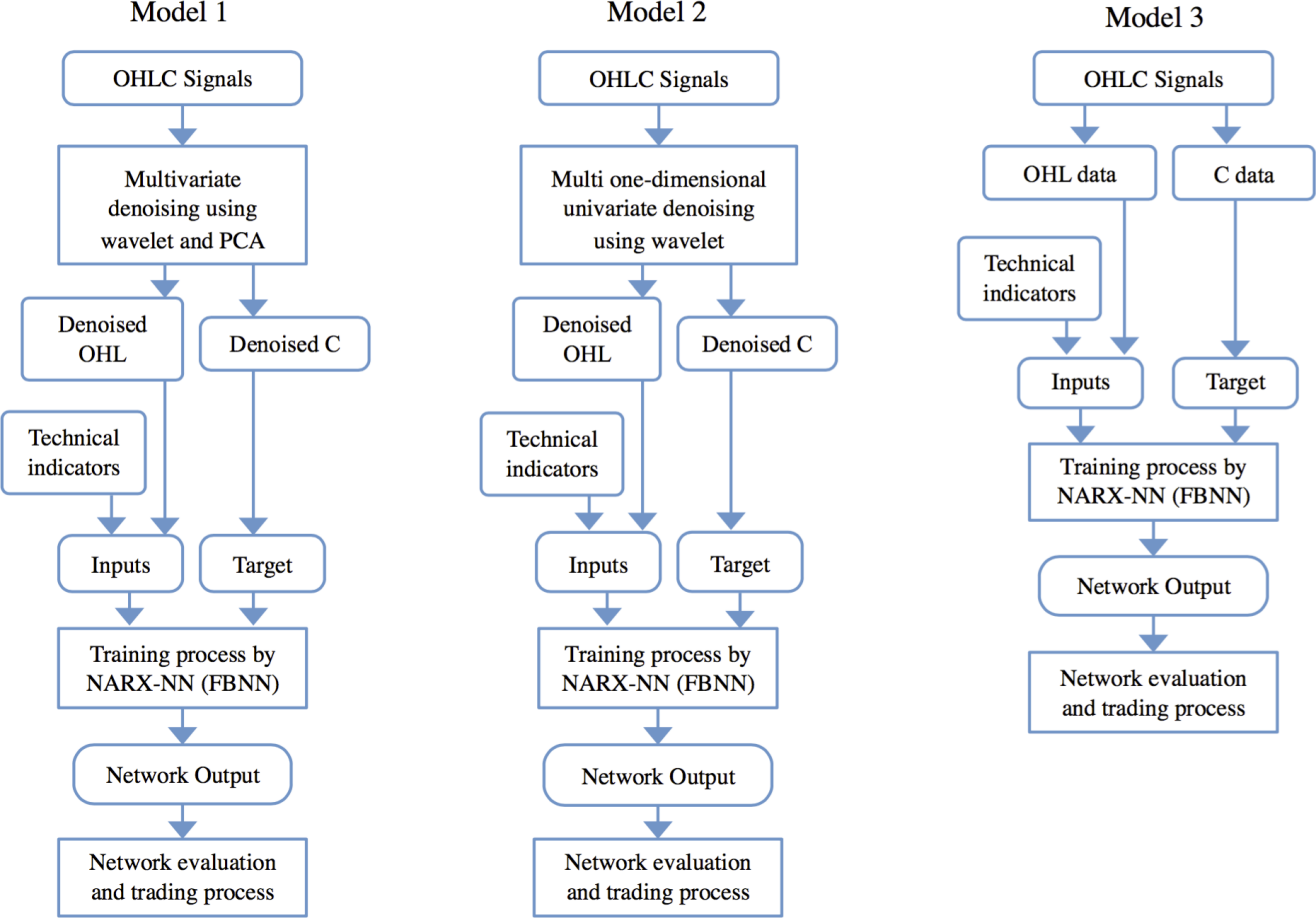
Architecture of the proposed models.

Fig 3 illustrates the proposed model architectures with a flowchart. Model 1, WPCA-NN, proposes a multivariate denoising by WPCA on the first part of each dataset on the OHLC signals to gain denoised OHLC signals. This process consists of various settings and variables as follows: wavelet type, level of denoising, thresholding strategy, and choosing the number of principal components, which are all discussed in the next section. Then, reconstructed denoised open, high, and low index (OHL signals) with the selected technical indicators as inputs and denoised close index (C signal) as targets are fed to the NARX-NN to train the network with the Levenberg-Marquardt algorithm. This procedure requires certain variables and settings as follows: number of delays, number of hidden layers and portions of training, validation, and testing, which are discussed in the next section. The trained networks are used to forecast the second part, the current quarter, with a one-step-ahead prediction technique. In this technique, the data of the first day of the second part will be added to the data of part 1, and then denoising process will be applied again. After that, the currently trained network will forecast the next day of part 2, based on the newly entered data. The predicted value (output) will be compared with the actual data (target value), to calculate the forecasting error and get signals for buy or sell, which will be discussed later. This process continues daily over the next 3 months to evaluate the predictive performance of that dataset. This procedure is repeated over all the markets.

Model 2, W-NARX-NN or WNN [18], differs from model 1 only in the denoising process. Denoising in this model is performed as multiple univariate denoising by wavelet separately on each component of the OHLC signal. This procedure requires some variables and settings, including wavelet type, level of denoising, and thresholding strategy. The rest of the process follows as with model 1.

Model 3 is a pure NARX-NN [45, 46] with no denoising as preprocessing of data undertaken in either the training or evaluation steps. OHLC signals and the technical indicators are directly fed to the NARX-NN and the procedure continues in the same way as for the rest of the models for both the training and evaluation parts. In conclusion, both ensemble and single models require different variables and settings in their structure, as discussed in the next section.

The key feature of a successful hybrid model is the settings of its structural elements, which are wavelet type, level of denoising, thresholding strategies, and the selection of the number of principal components. To perform a Wavelet Transform in the preprocessing stage, it is necessary to select the proper wavelet type from the range of strategies and various sequences available; these are Haar, Coiflets, DMeyer, Daubechies, and Symlets [60, 61]. Table 1 shows the wavelet families and their subsets used in this study to decompose the original OHLC signals. This study compares the performances of the various wavelets on each futures contract in order to obtain best-performing settings. Since each futures contract has different characteristics, each requires different decomposition techniques to be preprocessed adequately. Although Daubechies and Symlets use future data for transformations, causing boundary problems, we overcome this effect by time adjustments in the evaluation part. To overcome the boundary problem of these wavelet families, we input the data from the start to the $n_{i}^{th}$ data point into the model in order to forecast the $n_{i+1}^{th}$ index.

**Table 1:** Wavelet families and subsets.

Wavelet name	Subsets*
Haar	Haar
Daubechies	db2 db3 db4 db5 db6 db7 db8 db9 db10
Symlets	sym2 sym3 sym4 sym5 sym6 sym7 sym8
Coiflets	coif1 coif2 coif3 coif4 coif5
DMeyer	Dmeyer

* Subsets are presented in their shortened form as been appeared in wmulden function [52].

The next variable to select in preprocessing the Wavelet Transform is the level of decomposition, which is the number of times that the original signal is decomposed by wavelet transform. Aminghafari et al. [32] propose the maximum number of decompositions, 8 levels, and a rule to select the best level for decomposition. Although with more decomposition we may remove more noise and so obtain the underlying trend of the time series, we may also remove fluctuations that carry market characteristics. Hence, this research experiments with 1 to 8 levels of decomposition to find the optimal denoising level for each of the futures markets studied.

After using each wavelet transform on the original signals, we need to select denoising parameters. The first step is to select a thresholding method. The idea is to use the basis information that the wavelet coefficients propose: intuitively, small wavelet coefficients are combined with noise, while large wavelet coefficients contain more signal information than noise [18]. In this situation, it is rational to attain a suitable denoising of a given signal if we execute two basic processes: remove, in the wavelet picture, those components with small coefficients; and reduce the influence of components with large coefficients. Generally, all we are doing is thresholding or shrinking the absolute value of wavelet coefficients by a suitable method or rule such as: fixed form threshold, Rigorous SURE (Stein’s Unbiased Risk Estimate), Heuristic SURE, Minimax, Penalized high, Penalized Medium, or Penalized Low [33, 61]. We apply the mentioned thresholding rules on each set of decomposed signals to estimate the noise covariance matrix.

After the wavelet decomposes each of the original OHLC signals separately, PCA then analyzes these four signals simultaneously in order to extract the similar noise contained in all four signals and obtain the principal signals (denoised OHLC).

The final step in this preprocessing stage is to select the appropriate number of useful principal components. PCA is the common term for a method that uses complex fundamental mathematical principles to convert a number of feasibly correlated variables into a lesser number of linearly uncorrelated variables known as principal components. Although PCA has a variety of usages, it is generally applied in multivariate data analysis [32, 40]. This transformation is expressed in such a way that the initial principal component has the highest probability variance and each following component in turn has the largest variance possible, with the limitation that it is orthogonal to the earlier components. As Aminghafari et al. [32] suggest, the Kaiser criterion can be employed to select those components with matching eigenvalues larger than the mean of all the eigenvalues [54]. Implementing the Kaiser criterion, implanted as a module in the coded function of wmulden in Matlab [52], results in one principal component in model 1 analysis for all markets. Since OHLC consists of four signals, if we set four principal components in the study, it is equivalent to not applying the PCA technique; this is called model 2.

**Fig 4:**
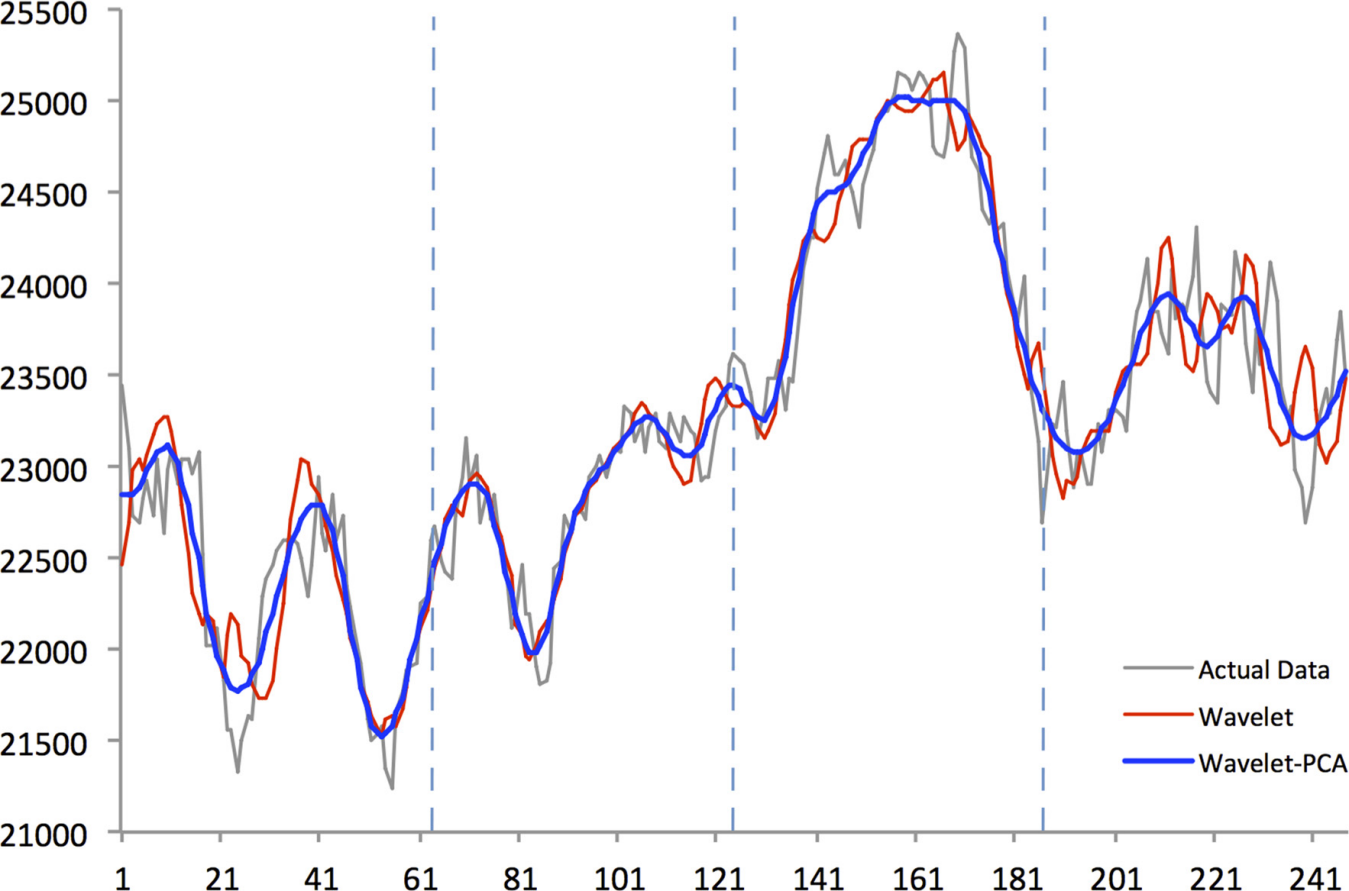
Univariate (Wavelet) and Multivariate (Wavelet-PCA) Denoising of Hang Seng futures 2014.

**Fig 5:**
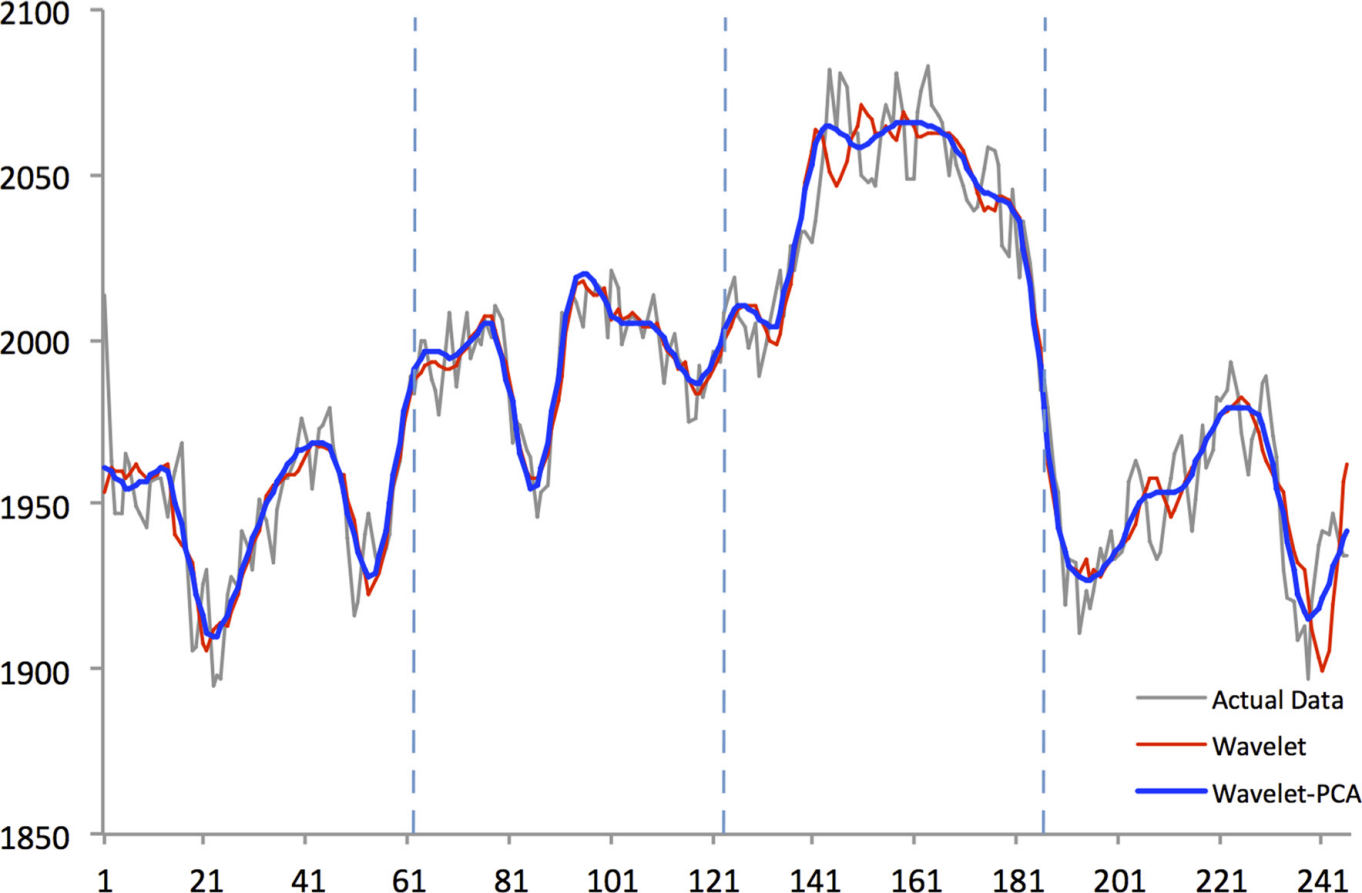
Univariate (Wavelet) and Multivariate (Wavelet-PCA) Denoising of KOSPI 200 futures 2014.

**Fig 6:**
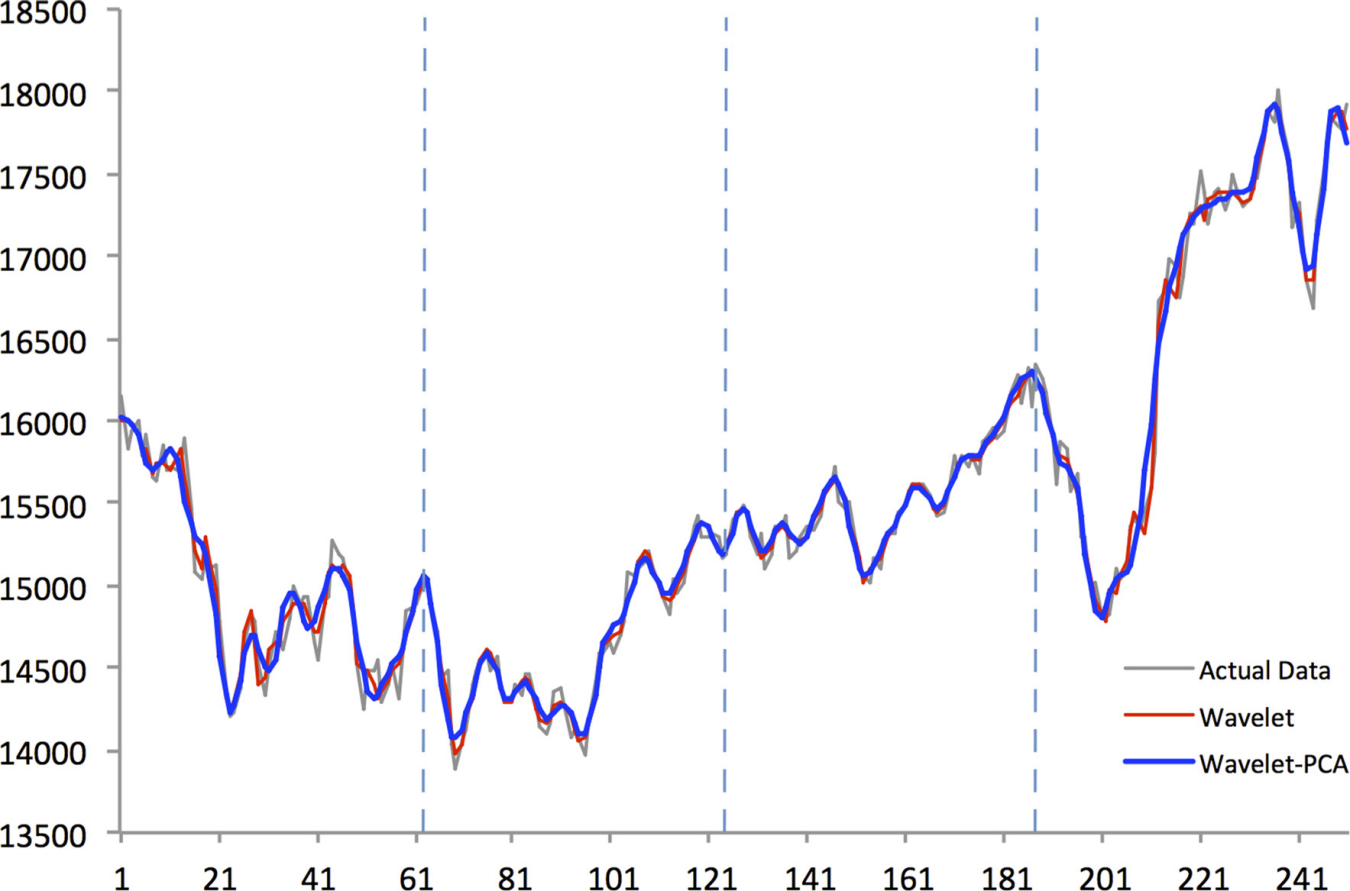
Univariate (Wavelet) and Multivariate (Wavelet-PCA) Denoising of NIKKEI 225 futures 2014.

**Fig 7:**
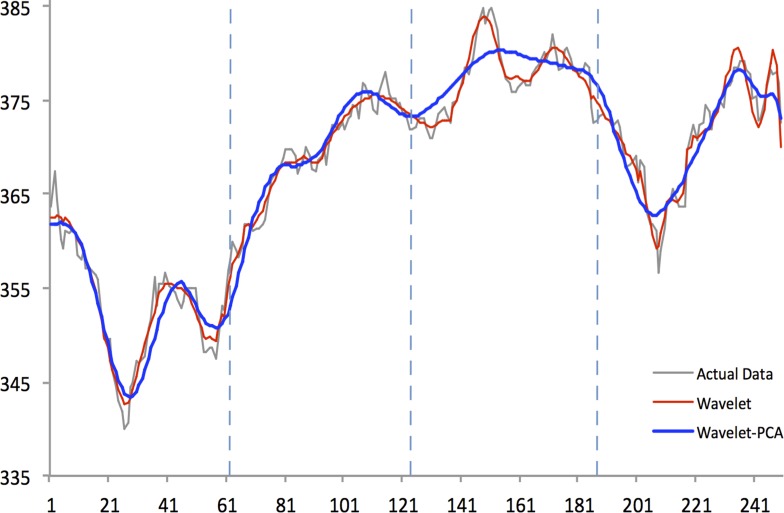
Univariate (Wavelet) and Multivariate (Wavelet-PCA) Denoising of SiMSCI futures 2014.

**Fig 8:**
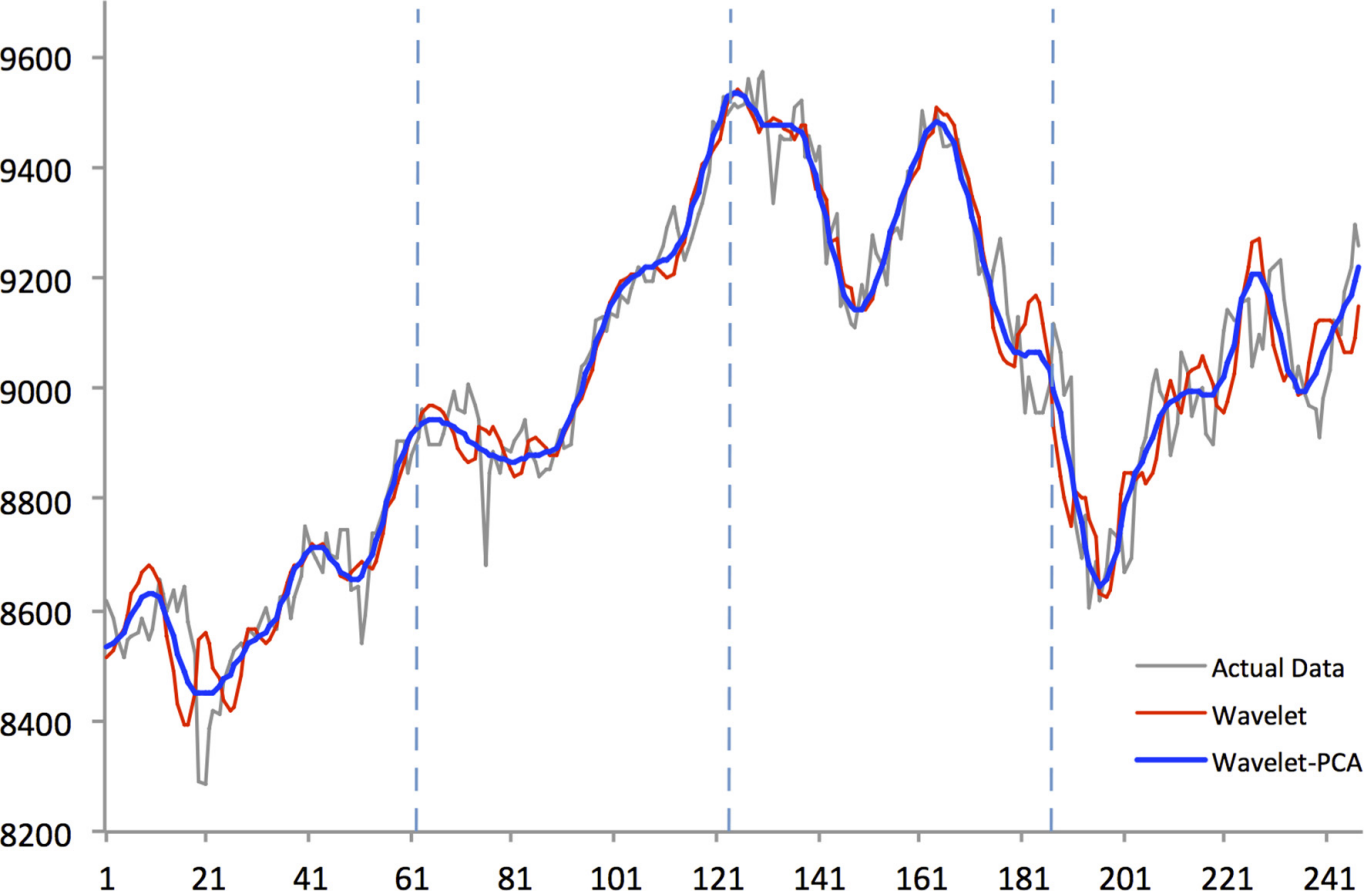
Univariate (Wavelet) and Multivariate (Wavelet-PCA) Denoising of TAIEX futures 2014.

Figs 4–8 show actual close data, generalized univariate wavelet denoising of close data, and multivariate denoising of OHLC using Wavelet-PCA on the Hang Seng, KOSPI 200, NIKKEI 225, SiMSCI, and TAIEX futures indices for 2014. S1–S5 Files illustrate results of the mentioned denoising process for all futures markets from 2002 to 2013. These two denoising are preprocessing for training part. The wavelet settings used for denoising in each market are based on the best-performing settings as shown in next section. According to the figures, although the level of decomposition and thresholding strategy for both univariate and multivariate denoising are the same, Wavelet-PCA seems to extract more noise than univariate wavelet and to achieve a more smoothed version of the original data. Hence, we may get better forecasting results from the underlying functions derived by Wavelet-PCA.

**Fig 9:**
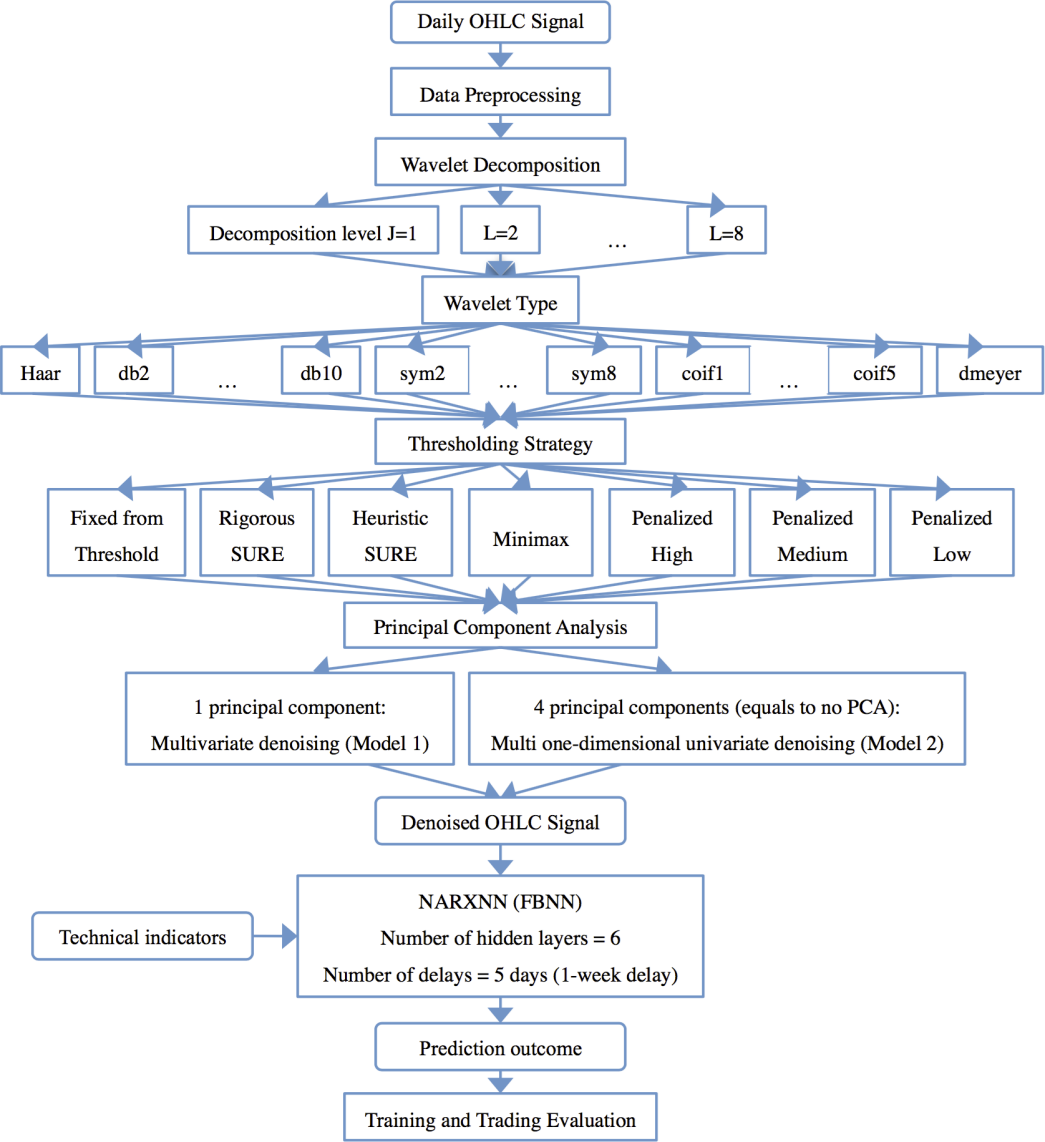
Research framework for models 1 and 2.

Practically, we perform a “loop” of the wavelet-PCA denoising method to achieve a set of all denoised signals in order to apply them as part of the input variables for ANN. Fig 9 illustrates this loop methodology for denoising and training the raw data with all mentioned settings of model 1, WPCA-NN, and model 2, WNN. This study uses 23 different wavelet settings (Table 1), 1–8 levels of decomposition, 7 different thresholding strategies, and 2 different PCA settings (one principal component for WPCA and four principal components for WNN) to achieve a total of 2,576 sets of denoised signals (denoised Open, denoised High, denoised Low, denoised Close).

Training a neural network with Levenberg-Marquardt needs two main configurations: number of hidden layers *h* and delays *d*. Although there are some techniques to choose suitable numbers of *h* and *d*, there may be no flawless rule of thumb and the best thing to do is to apply a backward elimination technique to achieve the best generalization result [17]. Backward elimination may be computationally expensive, but is reliable. With the range of results derived via the backward elimination technique, networks constructed with five days’ delay and six hidden nodes repeatedly achieve satisfactory results in all selected markets. There may be other combinations of *h* and *d* that perform better in a specific market, but since the main objective of this paper is studying the performance of the different denoising models, the research has been done repeatedly with one successful setting of ANN among all markets.

In this study, the Input series *x*(*t*) into NARX-NN is denoised open-high-low signals together with technical indicators, OHLC, RSI, MACD, MACD Signal, Stochastic Fast %K, Stochastic Slow %K, Stochastic %D, and Ultimate Oscillator calculated by original OHLC signals; while *y*(*t*) is the denoised close of the futures time series, which is considered as the target to be predicted. The prediction procedure is implemented with various settings of Wavelet PCA and NARX-NN, and the predictive performance is examined by NN error terms over the evaluation periods.

### Forecasting Performance

The Mean Absolute Percentage Error is applied as a measure of predictive accuracy to select which model performs the best [62]. MAPE can evaluate and compare the predictive power of the models. The definition of MAPE is

$$MAPE=\frac{\sum_{t=1}^{n}\left|\frac{y_{t}-y_{t}^{*}}{y_{t}}\right|}{n}$$ (16)

where *y*_*t*_ is the target value and $y_{t}^{*}$ is the predicted value. A lower MAPE value indicates better performance of the network, but we cannot expect it to be very close to zero, because financial markets are so volatile and fluctuate so widely. We select MAPE among other measures of prediction accuracy because its result is measured as a relative percentage, unlike other criteria such as root mean squared error and mean absolute error, which are biased to the scale of the index [28]. Hence, the accuracy of the offered models will be comparable across the five futures markets even with their different scale of indices.

### Profitability (Return) Performance

To check the profitability of a model in addition to its predictive accuracy, we establish a buy-and-sell trading rule strategy, which is widely used for profitability performance [63]. The strategy buys when the next period predicted value (target) is larger than the current market close and sells when the next period predicted value is smaller than the current market close:

$$y^{*}\left(t+1\right)>y\left(t\right):\;buy$$

$$y^{*}\left(t+1\right)<y\left(t\right):\;sell$$

where *y*(*t*) is the current market close and y*(*t* + 1) is the predicted value for the following market day. The net gain or loss is calculated every quarter during the second part of the evaluation period. Return of a trading strategy is widely considered as profitability performance of a model [63–65]. Hence, the summed return of this trading rule can be calculated by the following equation and used as a comparison scale among the models and markets:

$$Return\;\left(\%\right)\;=\;100\times \left(\sum_{t=1}^{b}(\frac{y_{t+1}-y_{t}}{y_{t}})+\sum_{t=1}^{s}(\frac{y_{t}-y_{t+1}}{y_{t}})\right)$$ (17)

where *b* denotes the total number of days for buying and *s* represents the total number of days for selling futures.

## Results

This research tests the forecasting ability based on MAPE and the return profitability of WPCA-NN on five Asian markets and compares the results to the existing methods such as NN [45, 46] and WNN [18] as well as to the threshold buy-and-hold [5] values. Performances of the different models are measured and evaluated on the basis of lowest MAPE and highest return values. After 2,576 simulations on the five Asian markets, the following settings of the best-performing networks are compiled in Table 2.

**Table 2:** Settings of the best-performing networks.

Settings	Hang Seng	KOSPI	NIKKEI	SiMSCI	TAIEX
Wavelet Name	coif5	sym6 or db9	coif5	db7	db9
Decomposition Level	3	3 or 4	2	4	2
Threshold	Penalized high	Penalized high	Penalized high	Penalized high	Penalized high
Principal Components	1	1	1	1	1
NARX-NN Delays	5	5	5	5	5
NARX-NN Hidden Layers	6	6	6	6	6
NARX-NN Training Period	3 years	3 years	3 years	3 years	3 years

The following results reported for each of the futures markets are based on these settings.

### Hang Seng Futures

Based on the HANG SENG futures results presented in Table 3, trained networks for all models are valid, as their MAPE values are quite low and acceptable. WPCA-NN outperforms WNN and NN over the whole testing period (4.06<15.44<579.4), the lesser MAPE ratio the better. Moreover, WPCA-NN gains the highest return among the models and a buy-and-hold strategy (34.7>27.9>12.5>8.5), based on the results shown in Table 4. NN performs very poorly, with an error term of 579.4% compared with the other models, so that its results are not acceptable. However, results of WPCA-NN and WNN are valid from 2005 to 2014. According to the best-performing networks in HANG SENG futures (Table 2), 3 levels of decomposition, coif5 wavelet, and penalized high thresholding strategy with application of WPCA-NN achieve significantly high performance and excess return. Moreover, Necula [66] applied a generalized hyperbolic distribution to forecast the Hang Seng index and achieved 5.3% yearly return. Huang et al. [67] performed a hierarchical coevolutionary fuzzy predictive model and gained 14.25% return.

**Table 3:** Performance of the models, MAPE ratio of evaluation results for HANG SENG futures.

Models	MAPE (%)	2005	2006	2007	2008	2009	2010	2011	2012	2013	2014	Average
WPCA-NN	Evaluation	3.3*	3.72*	11*	3.929*	3.5*	3.342*	3.354*	2.711*	2.296*	3.495*	4.065*
WNN	Evaluation	3.386	5.588	51.25	41.552	11.646	8.257	16.391	5.729	5.095	5.533	**15.443**
NN	Evaluation	99.19	245.13	1539.3	1598.01	621.58	379.93	615.35	214.39	230.25	250.88	**579.4**

* The best performance among models

**Table 4:** Return of the models, results for HANG SENG futures.

Models	2005	2006	2007	2008	2009	2010	2011	2012	2013	2014	Average
WPCA-NN	54.9*	76.3*	35.5	23	10.1	3	10	41*	54.9*	38.5*	34.7*
WNN	51.3	52.4	131.8*	-26.1	15.5	-8	-1.7	25.3	31.2	7.3	**27.9**
NN	-8.5	-0.4	-27.8	50.7*	2.9	12.1*	10.9*	39.6	23.8	22.2	**12.5**
Buy and Hold	7.8	30.5	37.4	-43.4	40.5*	5	-19.4	22.1	1.3	3	**8.5**

* The highest return among models

### KOSPI Futures

According to the MAPE ratio evaluation in Table 5, WPCA-NN outperforms WNN and NN models (2.24<2.8<41.99) for KOSPI Futures. Moreover, profitability of WPCA-NN is also greater than WNN, NN, and buy-and-hold strategies (68.5>58.5>23>11), as shown in Table 6. Moreover, results of the networks of the WPCA-NN and WNN are valid and reliable for the period of study. Based on the best-performing networks in KOSPI results (Table 2), 3 or 4 levels of decomposition, sym6 or db9 wavelets, and penalized high thresholding strategy with application of WPCA-NN gain significantly high performance and excess return. Kim et al. [68] applied artificial neural network and case-based reasoning and gained yearly return of 40.9% on the KOSPI 200 index, while Lee et al. [69] achieved 28.57% using a real-time rule-based trading system.

**Table 5:** Performance of the models, MAPE ratio of evaluation results for KOSPI futures.

Models	MAPE (%)	2005	2006	2007	2008	2009	2010	2011	2012	2013	2014	Average
WPCA-NN	Evaluation	2.348	2.111*	2.621*	3.027*	2.27*	2.059*	2.876*	2.231	1.687	1.2*	2.243*
WNN	Evaluation	2.338*	2.429	3.637	3.374	3.019	3.157	4.542	2.22*	1.677*	1.652	**2.805**
NN	Evaluation	33.07	30.43	93.36	68.6	31.02	27.42	89.56	23.73	14.37	8.3	**41.99**

* The best performance among models

**Table 6:** Return of the models, results for KOSPI futures.

Models	2005	2006	2007	2008	2009	2010	2011	2012	2013	2014	Average
WPCA-NN	98.9*	89	77.3*	85.9*	77.5*	38.2	83.7*	57.3	41.5*	35.5	68.5*
WNN	69.1	99.9*	67.1	22.2	77	43*	67.7	65*	40.8	36.5*	**58.8**
NN	26.7	22.9	-4.1	48.8	28	26.9	18.7	16.2	21	24.6	**23**
Buy and Hold	52.7	4.8	29.2	-36.7	43.6	20	-8.9	7.6	1	-3.8	**11**

* The highest return among models

### NIKKEI Futures

Based on the outcomes presented in Tables 7 and 8, the NIKKEI futures market also confirms the superiority of WPCA-NN over WNN, NN, and buy-and-hold. WPCA-NN evaluation performance is the best (4.05<10.88<451.89) and its yearly return is the highest (48.2>38.3>13.4>8). Moreover, WPCA-NN and WNN are valid in training and evaluation periods and achieve considerably higher returns than NN and buy-and-hold strategies. According to the best-performing networks in NIKKEI futures (Table 2), 2 levels of decomposition, coif5 wavelet, and penalized high thresholding with application of WPCA-NN achieve considerably high performance and return. In addition to that, Leung [70] achieved 17.2% yearly return by discriminant analysis and 13.78% by multilayered feedforward neural network on forecasting the Nikkei index. Necula [66] applied a generalized hyperbolic distribution to model the Nikkei 225 and gained 8.6% yearly return.

**Table 7:** Performance of the models, MAPE ratio of evaluation results for NIKKEI 225 futures.

Models	MAPE (%)	2005	2006	2007	2008	2009	2010	2011	2012	2013	2014	Average
WPCA-NN	Evaluation	2.741*	3.233*	3.283*	8.439*	2.782*	2.724*	3.13*	3.187*	7.856*	3.124*	4.05*
WNN	Evaluation	11.512	7.673	6.31	29.03	6.036	3.362	6.389	3.463	26.013	9.05	**10.884**
NN	Evaluation	395.82	391.55	320.73	1378.62	228.21	211.1	277.77	128.94	814.43	371.72	**451.89**

* The best performance among models

**Table 8:** Return of the models, results for NIKKEI 225 futures.

Models	2005	2006	2007	2008	2009	2010	2011	2012	2013	2014	Average
WPCA-NN	32.4	53*	66.6*	73.5*	38.9	46.5*	39.1*	40.1*	49.7	42.2*	48.2*
WNN	33.6	19.2	60.1	71.3	47.2*	39.7	26.9	32.6	50.5*	1.7	**38.3**
NN	-36.1	45.4	28.5	5.5	9.1	25.7	-15.4	33.7	-0.1	37.7	**13.4**
Buy and Hold	42.4*	5.3	-11.1	-40.9	18.1	-1.6	-20.2	26.2	49.5	12.6	**8**

* The highest return among models

### Singapore’s MSCI (SiMSCI) Futures

According to the results of forecasting SiMSCI, shown in Tables 9 and 10, WPCA-NN performs better (1.14<1.79<7.26) than WNN and NN, respectively. Moreover, WPCA-NN gains more return (71.1>53.2>28.4>7.9) than WNN, NN, and buy-and-hold, respectively. Although NN performs more poorly than the other two models, all models achieve more return than a buy-and-hold strategy. Based on the best-performing networks in SiMSCI results (Table 2), 4 levels of decomposition, db7 wavelets, and penalized high thresholding strategy with application of WPCA-NN gain significantly high performance and return. Moreover, Quah & Srinivasan [71] performed a neural network model on forecasting the Singapore index and achieved 25.64% yearly return; Chiang & Doong [72] gained about 15% yearly return performing generalized autoregressive conditional heteroscedasticity.

**Table 9:** Performance of the models, MAPE ratio of evaluation results for SiMSCI futures.

Models	MAPE (%)	2005	2006	2007	2008	2009	2010	2011	2012	2013	2014	Average
WPCA-NN	Evaluation	0.886*	0.986	2.585	2.173*	0.959*	0.32*	0.551*	1.202*	0.508*	1.254*	1.142*
WNN	Evaluation	1.21	0.84*	2.164*	2.497	2.775	2.428	1.24	2.109	1.193	1.467	**1.792**
NN	Evaluation	3.05	6.7	21.64	17.59	6.24	3.4	6.49	3.26	2.37	1.87	**7.26**

* The best performance among models

**Table 10:** Profit of the models, results for SiMSCI futures.

Models	2005	2006	2007	2008	2009	2010	2011	2012	2013	2014	Average
WPCA-NN	47.4*	84.1*	125.3*	117.9*	75.2*	62.2*	75.6*	37.6	47.6*	38.5*	71.1*
WNN	36.4	81.1	114.4	78.7	55.4	45.4	57.4	25.6	17.9	20	**53.2**
NN	13.9	15.2	0.6	27.8	55.7	31.1	56.1	40.3*	15.5	28	**28.4**
Buy & Hold	16.2	33.5	-2.3	-45.8	58.9	16.2	-11.1	8.7	-0.7	5.9	**7.9**

* The highest return among models

### TAIEX Futures

Based on the TAIEX results presented in Tables 11 and 12, trained networks for all models are valid, as their MAPE values are less than 5%. Moreover, WPCA-NN outperforms WNN and NN (2.71<5.00<143.77) according to the MAPE evaluation and achieves more profit (45.4>35.4>8.7>7.7) according to the yearly return. According to the best-performing networks in TAIEX results (Table 2), 3 levels of decomposition, db9 wavelet, penalized high thresholding strategy, and a delay of one week with application of WPCA-NN, gain considerably high performance and return. Although these results are robust and valid in different evaluation subsets and years, they relate to the characteristics of the market and may change in the far future. Not only are we saying this combination is the most appropriate setting for TAIEX forecasting, but also that any other combination of these parameters performs comparably better than the others, as these parameters represent common characteristics of the market across 10 years. In addition to that, Cheng et al. [73] gained 15.07% average yearly return by applying a hybrid model based on rough sets theory and genetic algorithms on the TAIEX stock index. However, Hsieh et al. [28] achieved 21.84% yearly return on TAIEX by performing an integrated system of wavelet transforms and recurrent neural networks based on artificial bee colony algorithm.

**Table 11:** Performance of the models, MAPE ratio of evaluation results for TAIEX futures.

Models	MAPE (%)	2005	2006	2007	2008	2009	2010	2011	2012	2013	2014	Average
WPCA-NN	Evaluation	1.641*	2.826*	3.99*	4.537*	2.437*	2.325*	2.845*	2.689*	1.915*	1.921*	2.713*
WNN	Evaluation	2.778	3.629	9.1	7.911	3.745	3.459	9.358	3.141	3.328	3.598	**5.005**
NN	Evaluation	52.32	108.03	259.53	397.79	130.28	97.32	212.44	80.63	43.44	55.92	**143.77**

* The best performance among models

**Table 12:** Profit of the models, results for TAIEX futures.

Models	2005	2006	2007	2008	2009	2010	2011	2012	2013	2014	Average
WPCA-NN	49.8	44.2	25*	22.7*	60.6	55.1	58.6*	51.8*	47.9*	38.4*	45.4*
WNN	54.2*	49.1*	-8.4	6.9	42.8	57*	23	48.6	45.2	35.8	**35.4**
NN	0.3	0.1	-3.3	-9.8	15.5	37.9	-7	19.3	27.5	6.5	**8.7**
Buy and Hold	5.2	22.6	0.5	-39.8	71.4*	8.5	-20.6	8.1	12.6	8.1	**7.7**

* The highest return among models

**Fig 10:**
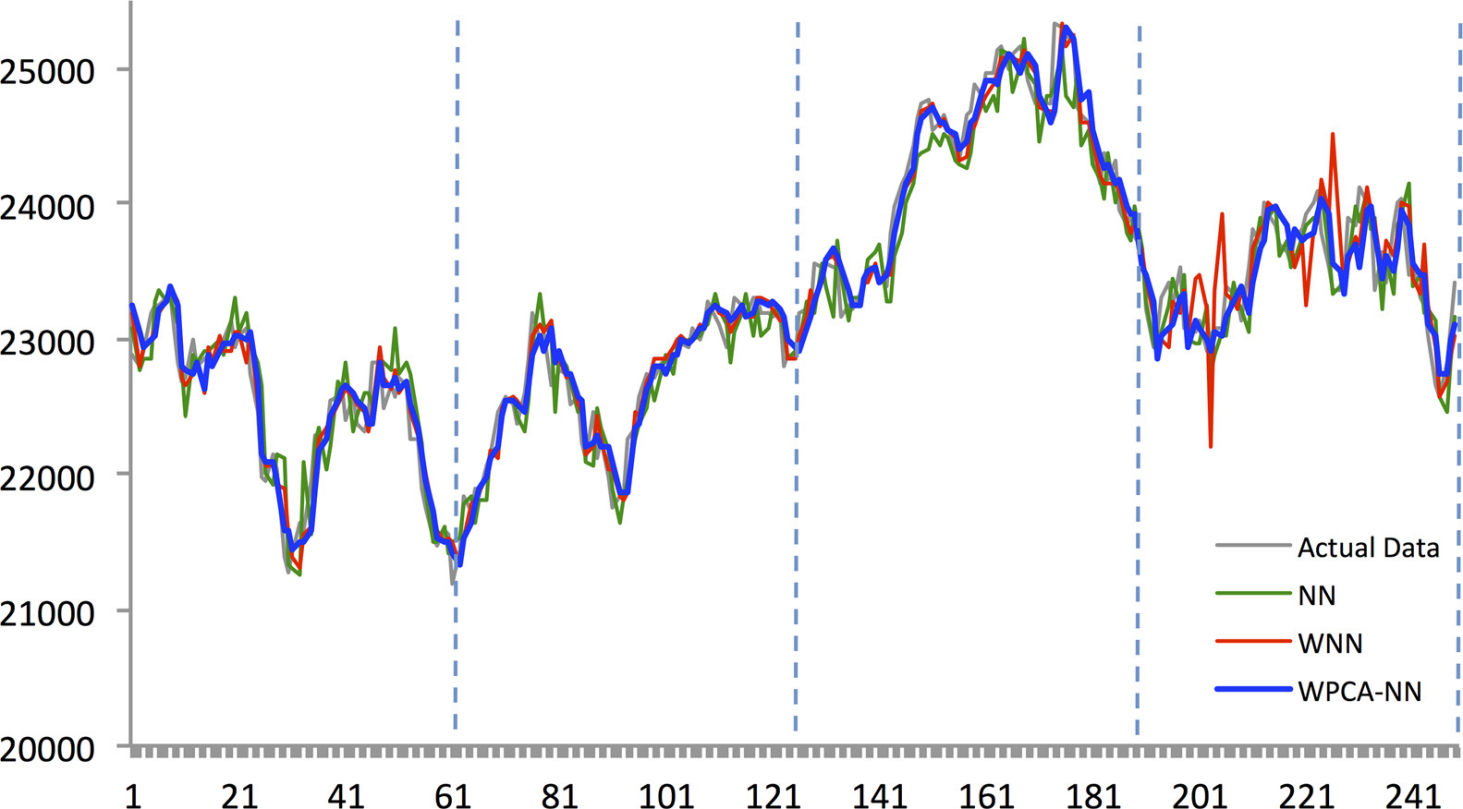
Forecasting results of all models for Hang Seng futures in 2014.

**Fig 11:**
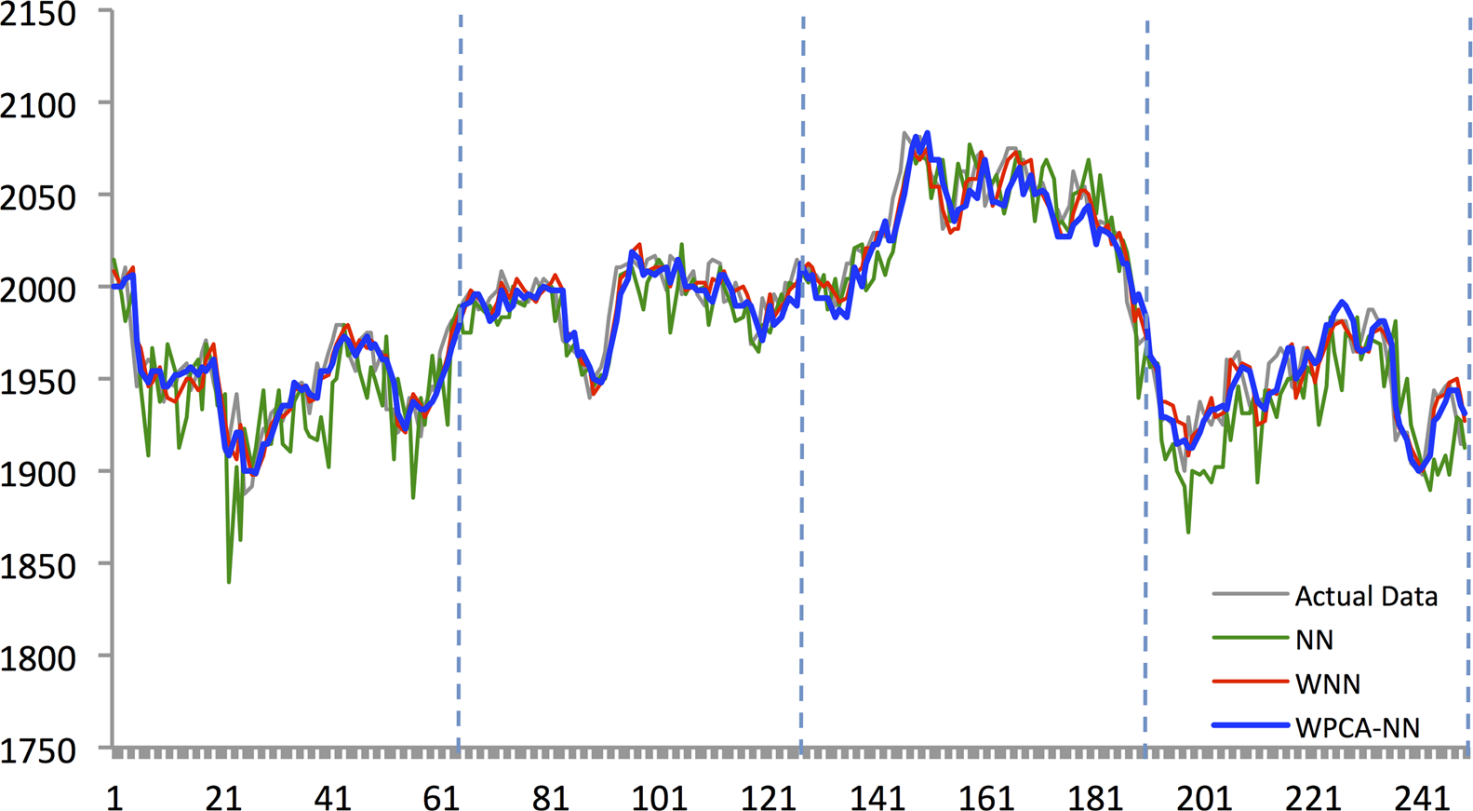
Forecasting results of all models for KOSPI 200 futures in 2014.

**Fig 12:**
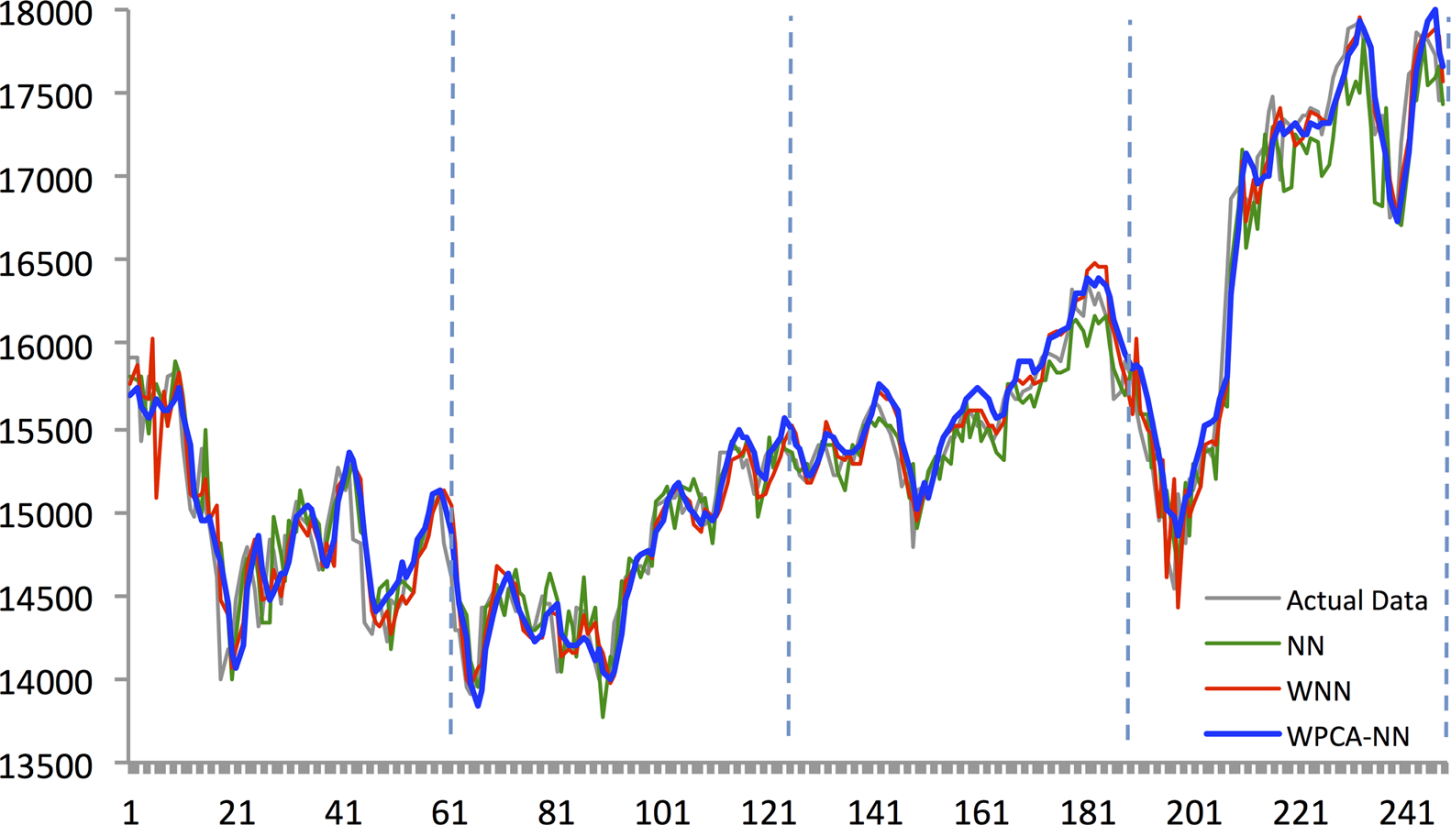
Forecasting results of all models for NIKKEI 225 futures in 2014.

**Fig 13:**
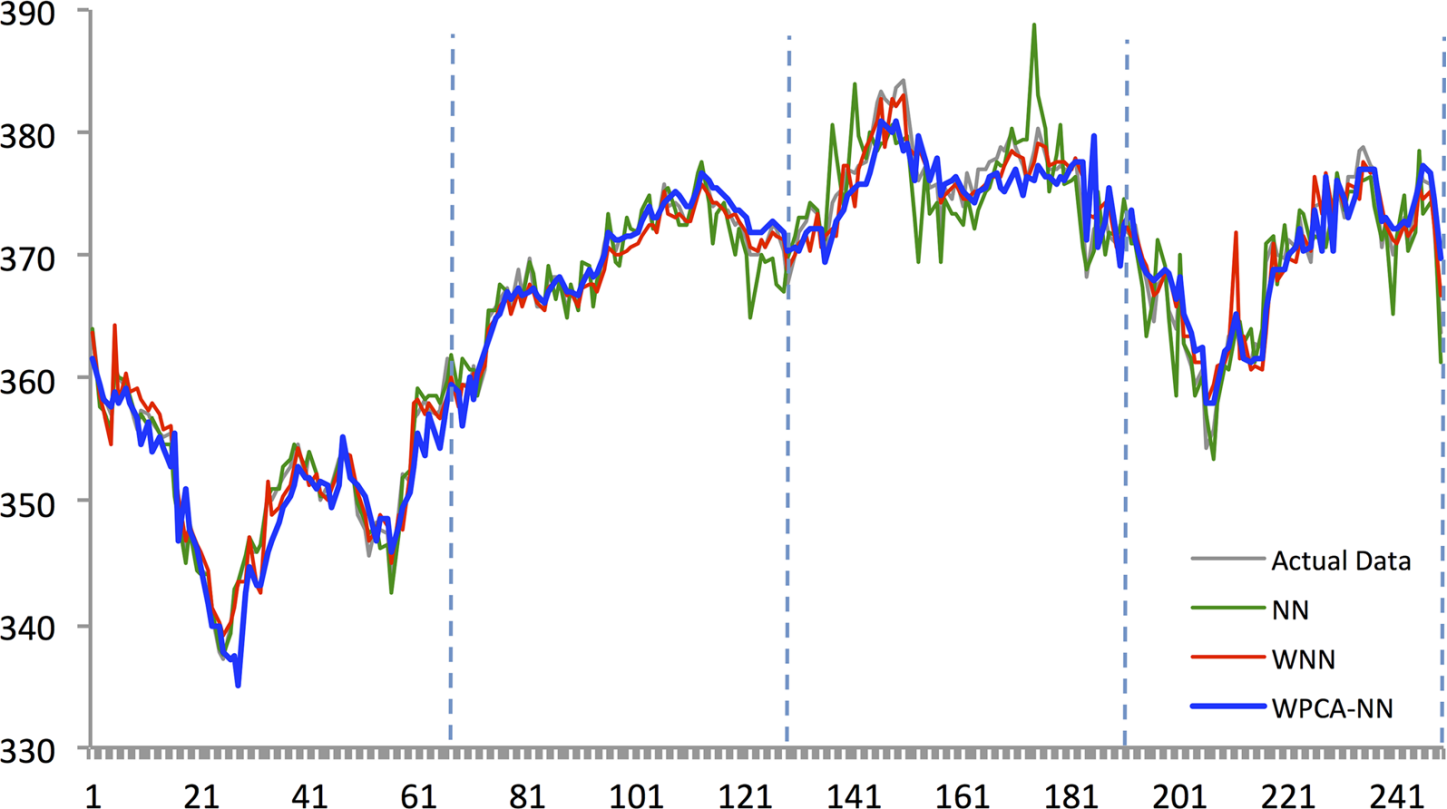
Forecasting results of all models for SiMSCI futures in 2014.

**Fig 14:**
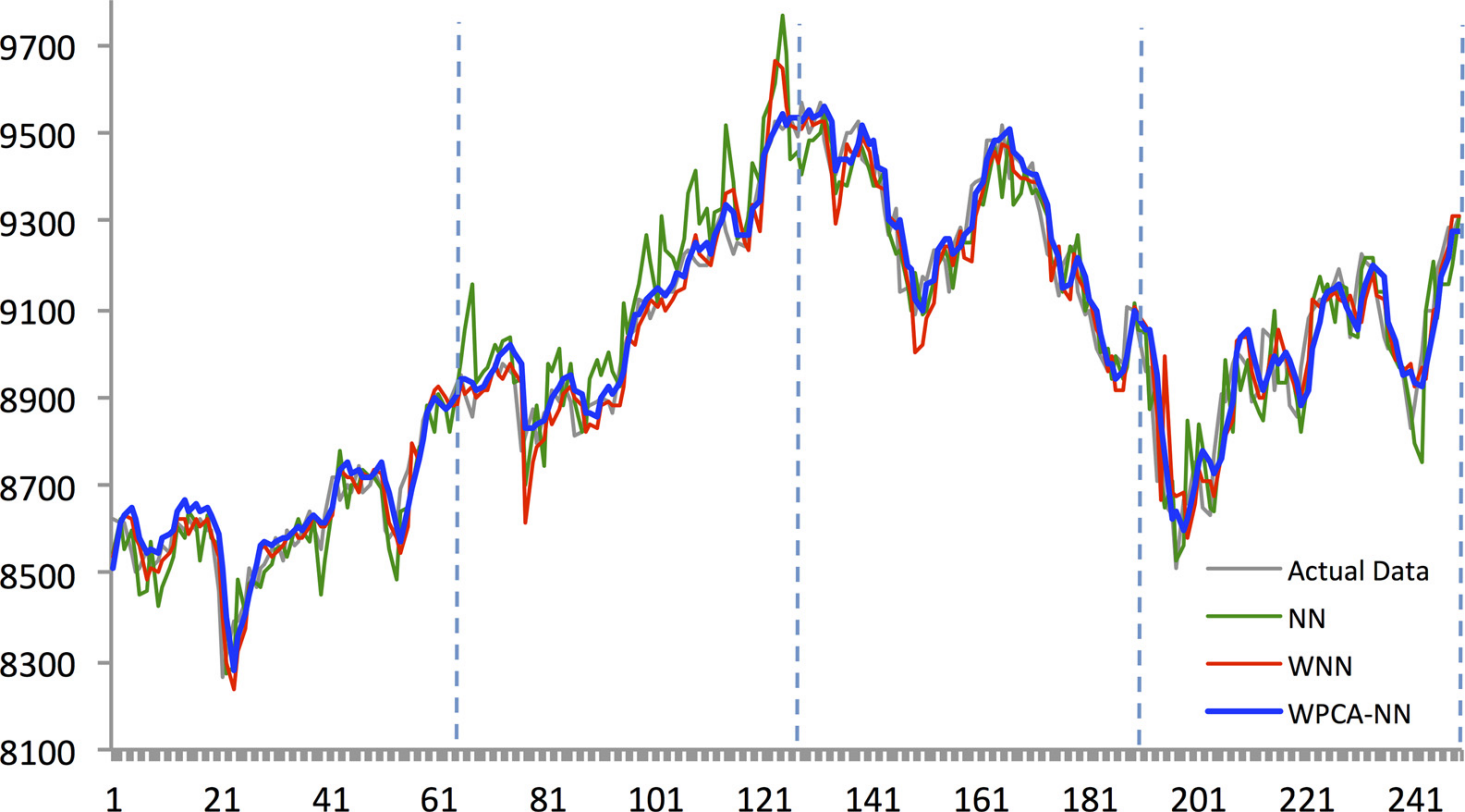
Forecasting results of all models for TAIEX futures in 2014.

Figs 10–14 show the results of WPCA-NN, WNN, and NN in one-step-ahead forecasting on TAIEX, SIMSCI, NIKKEI, KOSPI, and HANG SENG futures respectively for 2014, including the final four quarters of the forecasting period. The forecasting results of all quarters from 2005 to 2013 for all markets are illustrated in S6–S10 Files. According to the figures, NN is the most sensitive to fluctuations in the data, but WPCA-NN and WNN appear to be less sensitive to noise due to the application of wavelet transforms. As shown in the figures and Tables 2–11, not only are forecasting results of WPCA-NN and WNN more fitted to the actual data (lower MAPE values), but also they achieve higher excess return. However, a lower MAPE value, which means a higher performance and a better fitted forecasting outcome, does not ensure a higher return, and vice versa. Hence, selecting the best settings and network from the results is a bit problematic. This, therefore, could be a focus point for future studies. Having obtained the best settings for selected markets, we choose the most profitable networks with acceptable MAPE values. Moreover, as shown in the figures, WNN is more sensitive to fluctuations than WPCA-NN. In addition to that, WPCA-NN appears to result in a more smoothed version of the original signal. For some days, the forecasting results of WNN show a large difference and a wrong market direction compared with the actual data. However, on the same days, WPCA-NN recognizes the changing direction of the market much more accurately and creates better fitted predictions and higher return.

## Conclusion

This paper proposes a hybrid model, WPCA-NN, for futures price prediction; one that assembles several intelligent models and soft computing methods. This trading system is developed in four stages: (1) data preprocessing using wavelet analysis and PCA as multivariate denoising technique, which is applied to decompose the futures price time series in order to eliminate the same noise from OHLC signals; (2) use of some technical indicators and denoised OHL signals to construct the input series selected via a backward elimination technique; (3) application of the recurrent dynamic neural network; and (4) use of a simple trading strategy to gain more empirical results.

The proposed trading system, WPCA-NN, is compared with the existing methods such as Buy and Hold [5], pure recurrent dynamic neural network [45, 46], and WNN [18, 32],which is a generalized form of univariate denoising in multiple one-dimensional signals. In order to show that WPCA-NN is sufficiently robust, this trading system has been applied to five Asian futures markets, namely Hang Seng, KOSPI, NIKKEI 225, SiMSCI, and TAIEX futures for ten years (2005–2014). Simulation results indicate that a WPCA-NN trading system with multivariate denoising using Wavelet-PCA preprocessing and a recurrent dynamic neural network outperforms other models, NN and WNN as well as the threshold buy-and-hold in all five futures markets. WPCA-NN gains more return than other models including a buy-and-hold strategy in all examined futures markets in this period of time. On the other hand, multivariate denoising of WPCA-NN on OHLC enhances the denoising process and results in more accurate forecasting and a more profitable trading system. Additionally, the average returns using WPCA-NN are significantly higher than results from previous studies such as [28, 66–73]. Tables 13 and 14 show the summary of forecasting performance and profitability of the three models against buy-and-hold for the five East Asian futures markets. The proposed forecasting model is provided in S11 File as a Matlab script.

**Table 13:** Summary of forecasting performance, MAPE ratio, from 2005 to 2014.

Markets	WPCA-NN	WNN	NN
HANG SENG Futures	4.065	15.443	579.4
KOSPI 200 Futures	2.243	2.805	41.99
Nikkei 225 Futures	4.05	10.884	451.89
SiMSCI Futures	1.142	1.792	7.26
TAIEX Futures	2.713	5.005	143.77

**Table 14:** Summary of Average Annual Returns from 2005 to 2014.

Markets	WPCA-NN	WNN	NN	Buy-and-Hold
HANG SENG Futures	34.7	27.9	12.5	8.5
KOSPI 200 Futures	68.5	58.8	23	11
Nikkei 225 Futures	48.2	38.3	13.4	8
SiMSCI Futures	52.7	51.6	22	8.4
TAIEX Futures	45.4	35.4	8.7	7.7

The results are entirely consistent with other similar studies such as [74–77] on machine learning using technical analysis indicators, whereby frequent accurate predictions lead to abnormal returns. The findings of this research indicate that in view of the growing efficiency of rapidly growing financial markets that have moved beyond traditional technical analysis tools [8, 9], machine learning trading systems may be a novel profitable strategy to trade futures contracts. It can be concluded that a significant implication arising from the findings of this study is that at least for three months ahead, using the best settings obtained by repeated simulations of the previous three years, weary traders in Hang Seng, KOSPI, Nikkei 225, SiMSCI, and TAIEX futures can have a promising new trading system, WPCA-NN, to combat the volatile nature of these rapidly changing markets. Moreover, this model can be applied in other fields of study that include multivariate signals and require forecasting tools to expand the barriers of the science.

Although the proposed hybrid system achieves promising forecasting results, it still possesses some deficiencies, which means the approach may be expanded. In the future, a different intelligent ensemble model, such as a support vector machine (SVM) or adaptive neuro-fuzzy inference system (ANFIS) with different algorithms like Genetic or Bee Colony Algorithms, along with Wavelet-PCA, might be employed to financial time series forecasting problems. In addition, the selection of best-performing networks has two different destinies via statistical performance (e.g., MAPE value) and trading strategies (Return). Therefore, for future studies, analysis of both statistical and trading performance, and finding a more precise method to choose the best network and setting from the results, are suggested.

## Supporting Information

S1 File Univariate (wavelet) and multivariate (Wavelet-PCA) denoising of Hang Seng futures, 2002–2013. (PDF)

S2 File Univariate (wavelet) and multivariate (Wavelet-PCA) denoising of KOSPI 200 futures, 2002–2013. (PDF)

S3 File Univariate (wavelet) and multivariate (Wavelet-PCA) denoising of NIKKEI 225 futures, 2002–2013. (PDF)

S4 File Univariate (wavelet) and multivariate (Wavelet-PCA) denoising of SiMSCI futures, 2002–2013. (PDF)

S5 File Univariate (wavelet) and multivariate (Wavelet-PCA) denoising of TAIEX futures, 2002–2013. (PDF)

S6 File Forecasting results of all models for Hang Seng futures, 2005–2013. (PDF)

S7 File Forecasting results of all models for KOSPI 200 futures, 2005–2013. (PDF)

S8 File Forecasting results of all models for NIKKEI 225 futures, 2005–2013. (PDF)

S9 File Forecasting results of all models for SiMSCI futures, 2005–2013. (PDF)

S10 File Forecasting results of all models for TAIEX futures, 2005–2013. (PDF)

S11 File Programmed script for the proposed forecasting model supported by Matlab software. (ZIP)

S1 Table Results for unit root test. (PDF)

S2 Table Results for Johansen cointegration test. (PDF)

S3 Table Results for error correction model. (PDF)

S4 Table Results for serial correlation test. (PDF)
