# Supplementary material for: Forecasting East Asian Indices Futures via a Novel Hybrid of Wavelet-PCA Denoising and Artificial Neural Network Models
Source: PLoS One. 2016 Jun 1;11(6):e0156338. doi: 10.1371/journal.pone.0156338 (PMC4889155; doi:10.1371/journal.pone.0156338)

S1 file. Univariate (Wavelet) and Multivariate (Wavelet-PCA) Denoising of Hang Seng futures 2002-2013

Year 2002

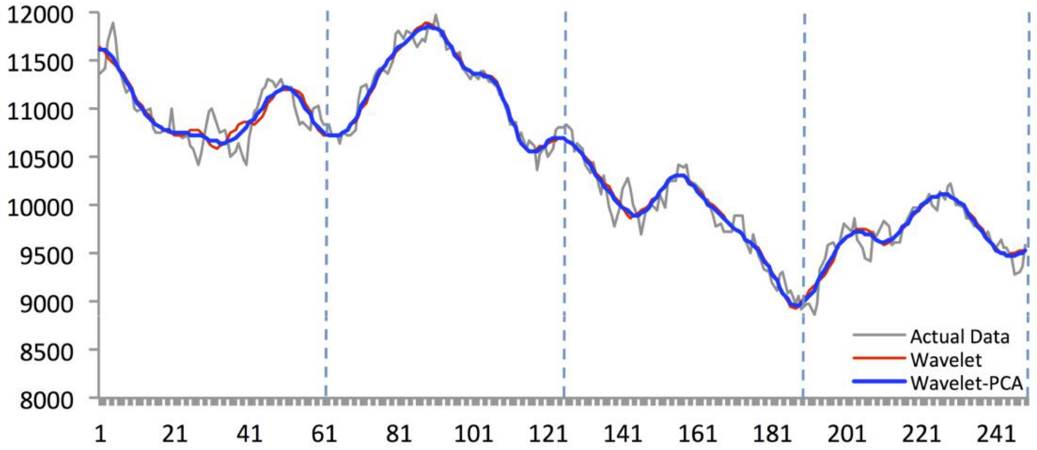

Year 2003

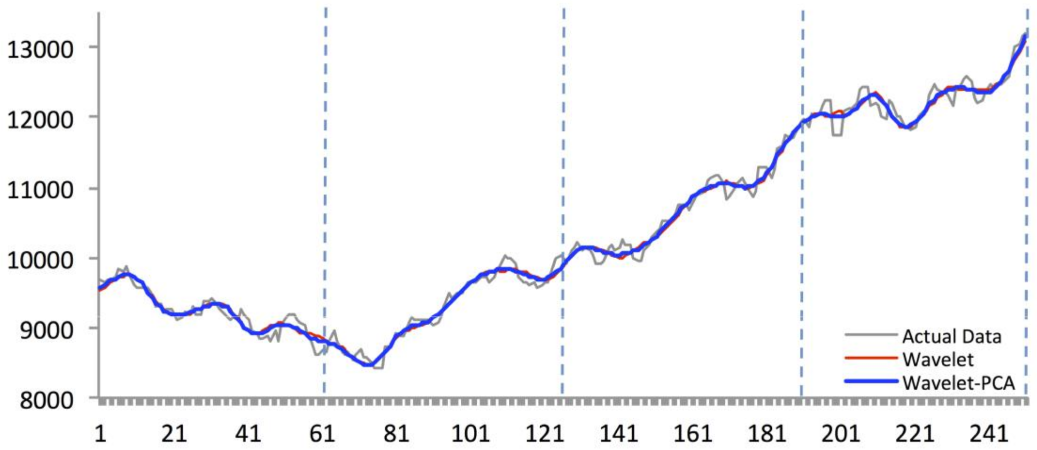

Year 2004

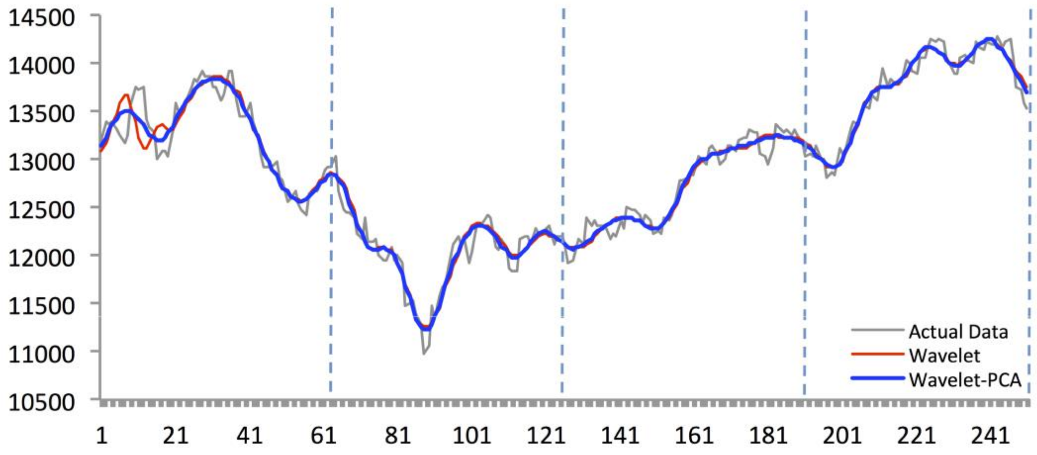

Year 2005

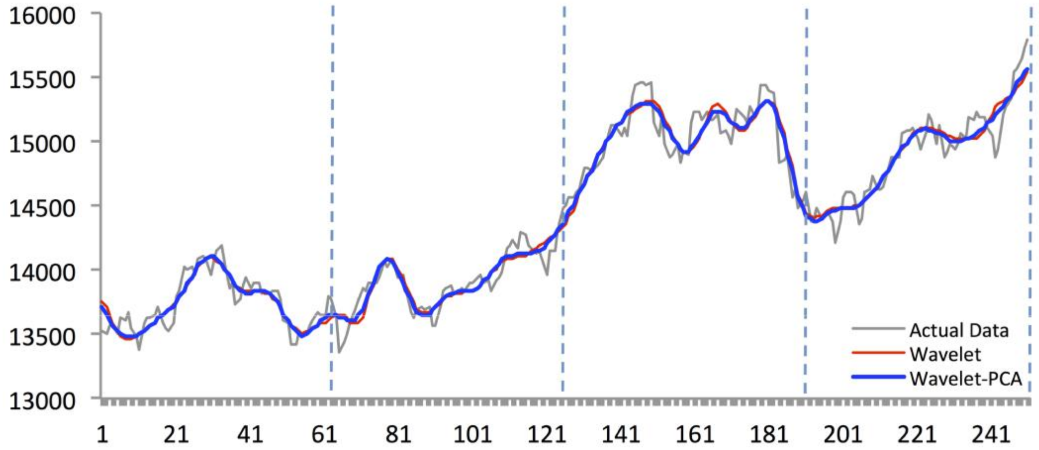

Year 2006

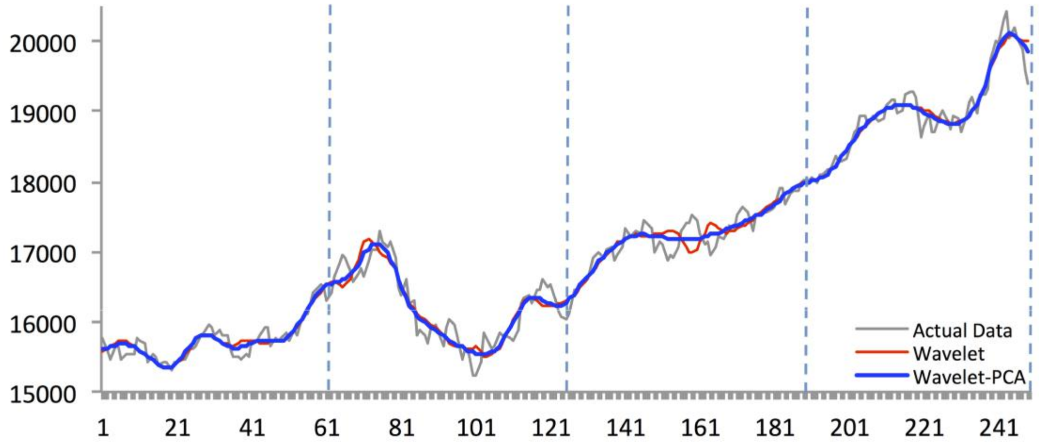

Year 2007

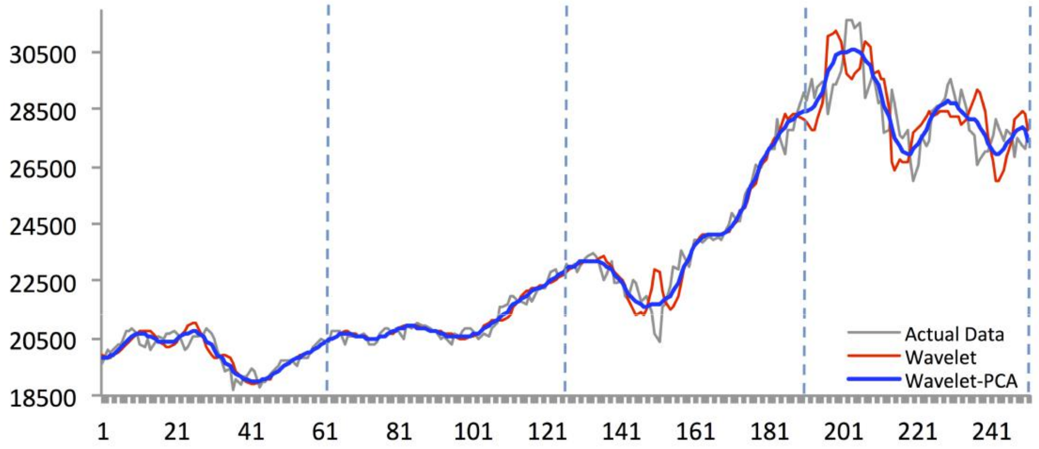

Year 2008

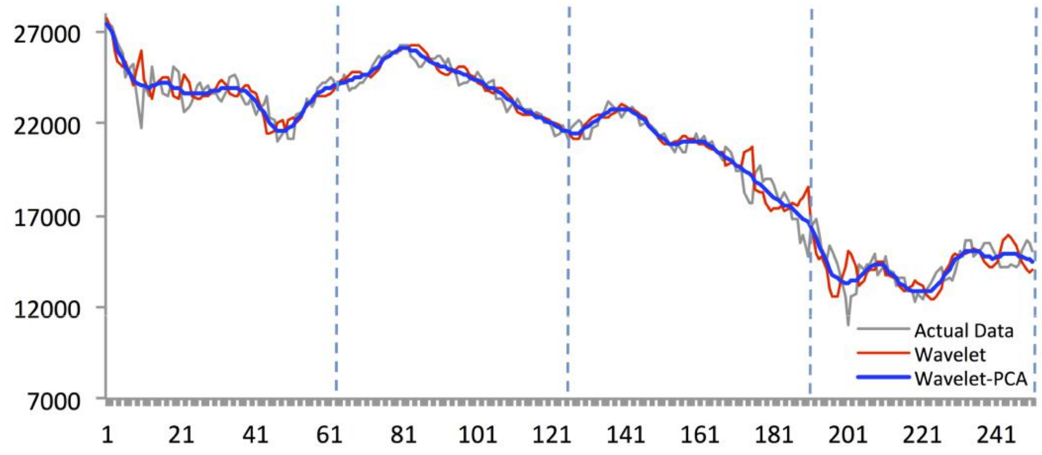

Year 2009

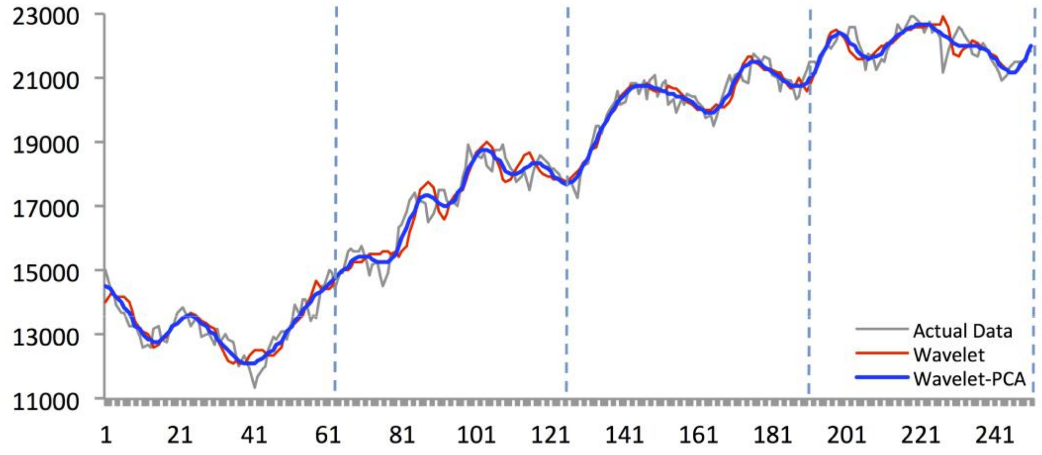

Year 2010

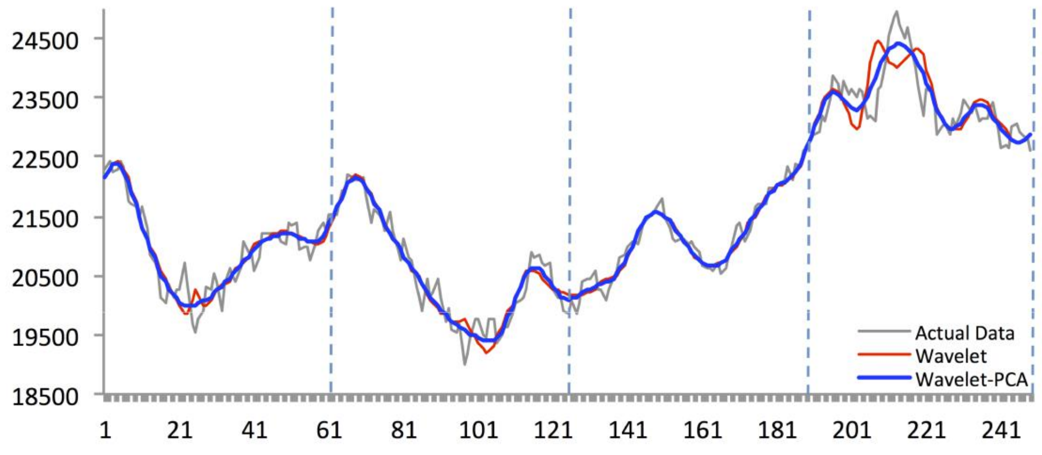

Year 2011

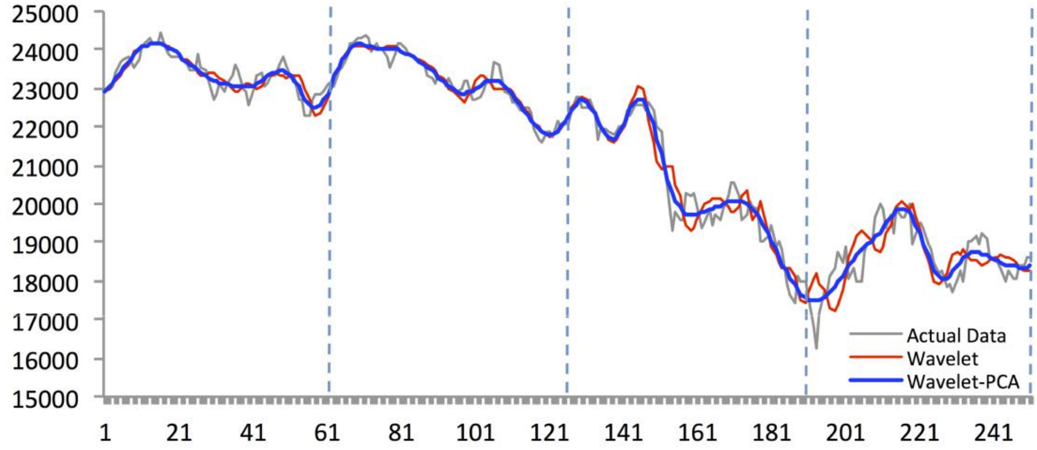

Year 2012

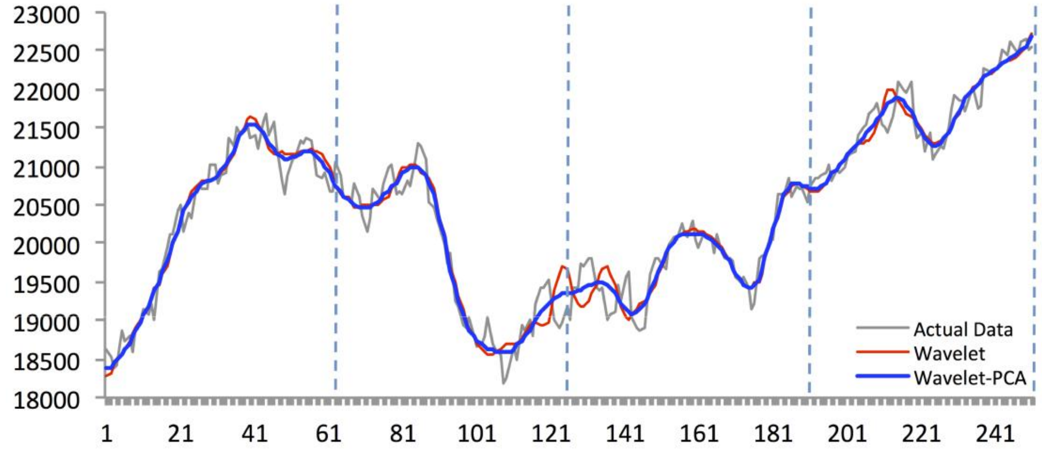

Year 2013

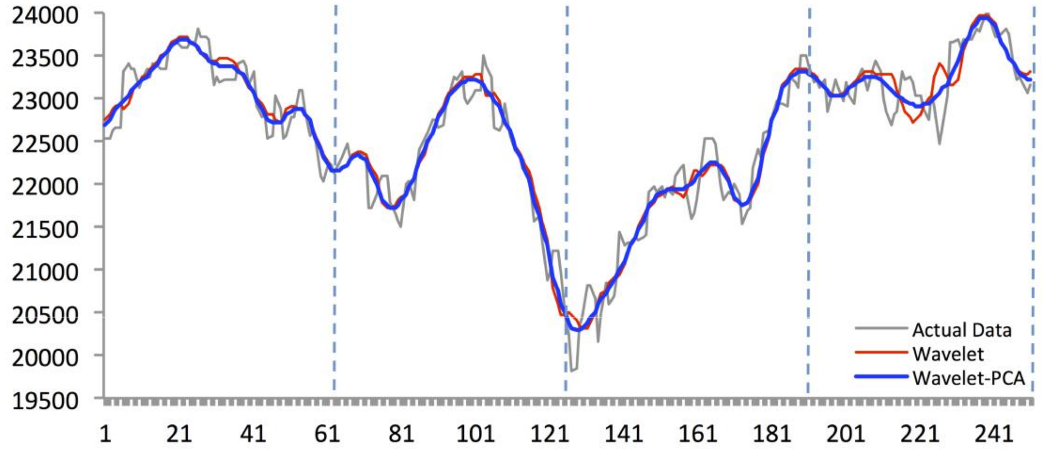

Supplement: S1 File — (PDF) [file pone.0156338.s001.pdf]
