# Supplementary material for: Forecasting East Asian Indices Futures via a Novel Hybrid of Wavelet-PCA Denoising and Artificial Neural Network Models
Source: PLoS One. 2016 Jun 1;11(6):e0156338. doi: 10.1371/journal.pone.0156338 (PMC4889155; doi:10.1371/journal.pone.0156338)
Supplement: S1 Table — (PDF) [file pone.0156338.s012.pdf]

S1A Table

Unit Root Test ADF, trend and intercept value

Markets	Test in the level form	The first difference form
HS futures	-2.59191	**-58.37271***
KOSPI 200	-2.491356	**-55.64235***
NIKKEI 225	-1.312561	**-58.40276***
SiMSCI	-1.889653	**-60.65505***
TAIEX futures	-2.76804	**-53.85111***

*Significant at 5% level
