# Supplementary material for: Forecasting East Asian Indices Futures via a Novel Hybrid of Wavelet-PCA Denoising and Artificial Neural Network Models
Source: PLoS One. 2016 Jun 1;11(6):e0156338. doi: 10.1371/journal.pone.0156338 (PMC4889155; doi:10.1371/journal.pone.0156338)
Supplement: S2 Table — (PDF) [file pone.0156338.s013.pdf]

S2 Table

Johansen Cointegration Test

Markets	Trace Statistic	Max-eigen Statistic
HS futures	4.935282*	4.935282*
KLCI futures	356.1845*	356.1845*
KOSPI 200	17.04305*	17.04305*
NIKKEI 225	23.86056*	23.86056*
SiMSCI	14.89672*	14.89672*
SNP500	7.576388*	7.576388*
TAIEX futures	6.027678*	6.027678*

*Significant at 5% level
