# Supplementary material for: Forecasting East Asian Indices Futures via a Novel Hybrid of Wavelet-PCA Denoising and Artificial Neural Network Models
Source: PLoS One. 2016 Jun 1;11(6):e0156338. doi: 10.1371/journal.pone.0156338 (PMC4889155; doi:10.1371/journal.pone.0156338)
Supplement: S3 Table — (PDF) [file pone.0156338.s014.pdf]

S3 Table

Markets	U(t-1) or Error Correction Term	Interpretation
Hang Seng futures	-0.165*	Long Run relationship between previous data and current data
KLCI futures	-0.371*	Long Run relationship between previous data and current data
KOSPI 200	-0.102*	Long Run relationship between previous data and current data
NIKKEI 225	-0.129*	Long Run relationship between previous data and current data
SiMSCI	-0.143*	Long Run relationship between previous data and current data
SNP500	-0.114*	Long Run relationship between previous data and current data
TAIEX futures	-0.114*	Long Run relationship between previous data and current data

*Significant in 5% confident
