# Supplementary material for: Forecasting East Asian Indices Futures via a Novel Hybrid of Wavelet-PCA Denoising and Artificial Neural Network Models
Source: PLoS One. 2016 Jun 1;11(6):e0156338. doi: 10.1371/journal.pone.0156338 (PMC4889155; doi:10.1371/journal.pone.0156338)
Supplement: S4 Table — (PDF) [file pone.0156338.s015.pdf]

S4 Table

Breusch-Godfrey Serial Correlation LM Test

Markets	Obs*R-squared	Prob. Chi-Square(5)
HS futures	8.050268	0.0535
KLCI futures	2.907285	0.0713
KOSPI 200	6.049925	0.0304
NIKKEI 225	3.00968	0.0695
SiMSCI	8.702567	0.0125
SNP500	6.388968	0.0472
TAIEX futures	11.51784	0.0542
